# Mechanical Response and Energy Absorption of Bio-Inspired Auxetic Hybrid Tubular Metamaterials

**DOI:** 10.3390/biomimetics11080556

**Published:** 2026-08-05

**Authors:** Sheng Huo, Fukun Xia, Shanqing Xu, Zhanyuan Gao, Dong Ruan

**Affiliations:** 1 School of Engineering, Swinburne University of Technology, Hawthorn, VIC 3122, Australia; shuo@swin.edu.au (S.H.); fxia@swin.edu.au (F.X.); sxu@swin.edu.au (S.X.); 2 School of Civil Engineering, Tianjin Chengjian University, Xiqing District, Tianjin 300384, China

**Keywords:** bio-inspired structures, auxetic tubular metamaterials, hybrid tubes, quasi-static compression, energy absorption, deformation mode

## Abstract

Bio-inspired auxetic–conventional hybrid tubular metamaterials were investigated for lightweight energy absorption. The tubes combined 6063 aluminium alloy inner tubes with 304 stainless-steel outer tubes containing oval, circular, rotating-square, or re-entrant perforations. Experiments showed that the outer tube altered the collapse mode of the inner tube and produced topology-dependent responses. The Circle-Hybrid tube achieved the highest mean specific energy absorption (SEA) of 6.95 ± 0.04 kJ/kg; the Oval-Hybrid tube was 17.1% lower at 5.76 ± 0.25 kJ/kg but had a 24.1% lower maximum force and a CFE 6.68 percentage points higher. Its SEA was approximately 159.5% higher than that of the Oval-Single tube. A validated finite element model was used to examine oval-hole aspect ratio and inner-tube wall thickness. At fixed porosity, decreasing the aspect ratio promoted progressive folding and increased load-bearing capacity and energy absorption, while an intermediate ratio maximised CFE. Increasing the inner-tube wall thickness enhanced load-bearing capacity and energy absorption but produced more localised or asymmetric buckling. These findings demonstrate that perforation topology can tailor collapse mode, peak-force demand, crushing efficiency, and energy absorption in hybrid tubes.

## 1. Introduction

**Figure 1:**
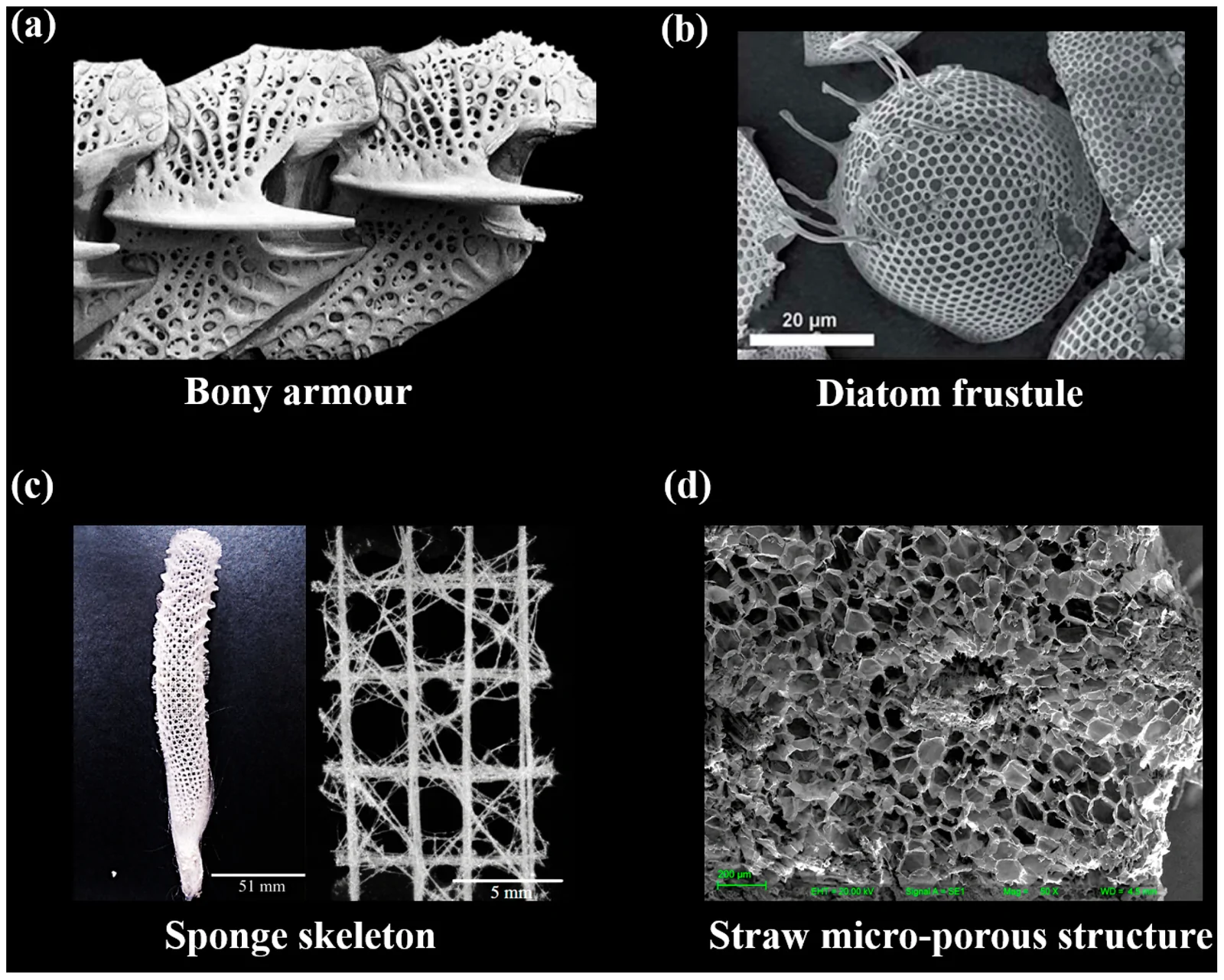
Representative porous and cellular biological architectures inspiring lightweight tubular energy absorbers: (**a**) porous bony armour of Hoplichthys haswelli [4]; (**b**) diatom frustule of Stephanopyxis turris [5]; (**c**) deep-sea glass sponge skeleton of Euplectella aspergillum with a hierarchical strut lattice [6]; and (**d**) straw micro-porous structure [7]. These examples illustrate porous, cellular, shell-like, hierarchical, and core-supported design principles relevant to deformation regulation and energy absorption. Panel (**a**) was adapted with permission from Ref. [4]. Copyright 2017, The Royal Swedish Academy of Sciences. Panel (**b**) was adapted and modified from Jantschke et al. [5], licensed under the Creative Commons Attribution 3.0 Unported Licence. Copyright © The Royal Society of Chemistry 2014. Panel (**c**) was adapted and modified from Robson Brown et al. [6], licensed under the Creative Commons Attribution 4.0 International Licence. Panel (**d**) was adapted and modified from Xu et al. [7], licensed under the Creative Commons Attribution 4.0 International Licence.

Natural and bio-inspired protective and load-bearing structures often achieve a balance between lightweight characteristics, mechanical resistance, and deformation adaptability through porous, cellular, hierarchical, or shell-like architectures [1,2,3,4,5,6,7,8]. Studies on cellular solids and engineered microlattice systems further show that such architectural features can regulate stiffness, load transfer, deformation stability, and energy absorption [9,10]. Representative examples include porous bony armour, diatom frustules, deep-sea glass sponge skeletons, and straw micro-porous structures (Figure 1). Although these natural and related bio-inspired systems differ in material composition, length scale, and biological function, they share a common mechanical principle: material is not distributed as a fully solid body, but is organised into pores, cells, ligaments, shells, struts, or core-supported forms to regulate stiffness, load transfer, buckling resistance, and deformation stability [5,6,7,8,9,10]. For instance, the porous bony armour of *Hoplichthys haswelli* provides a calcified protective morphology with distributed openings and pore-and-ligament features [4]. Diatom frustules, such as *Stephanopyxis turris*, possess ordered porous silica cell walls with honeycomb-like chambers and regular pore arrays, and diatom valves have been associated with mechanical strength and remarkable elastic moduli [5]. Similarly, the deep-sea glass sponge Euplectella aspergillum uses a hierarchical skeletal lattice with longitudinal, transverse, and diagonal struts, providing an efficient lightweight architecture for stiffness, buckling resistance, and load transfer [6,8]. Straw stems, such as sorghum and reed, contain microcellular and chamber-like porous structures that provide lightweight support and have been used as biomimetic prototypes for honeycomb energy-absorbing structures [7].

These biological examples highlight the mechanical relevance of porous, cellular, hierarchical, and core-supported architectures for lightweight structural design. Features such as controlled porosity, ligament continuity, shell-like load paths, diagonal reinforcement, microcellular chambers, and internal support can improve load transfer, buckling resistance, deformation stability, and energy dissipation [6,7,8,9,10]. In addition, the characteristic elastic–plateau–densification response of cellular solids provides an effective mechanism for energy absorption under compression [9].

Thin-walled tubes have been widely investigated as efficient energy absorbers in structural crashworthiness owing to their ability to dissipate energy under axial compression [11,12,13,14]. Their advantages, including low cost, lightweight characteristics, structural toughness, and ease of manufacture, make them attractive for aerospace, automotive, protective equipment, and civil infrastructure applications [15,16]. Recent bio-inspired studies have further demonstrated that biological concepts can be transferred into tubular or cellular structures to improve compressive performance and crashworthiness. For example, bio-inspired honeycomb and tubular structures based on plant, bamboo, or porous biological features have been shown to regulate deformation modes and enhance energy absorption under compression [1,17,18]. These studies indicate that the mechanical performance of lightweight energy absorbers depends not only on the material and global geometry, but also on how biological features are abstracted into engineering topologies.

Several structural strategies have been proposed to improve the energy absorption and collapse stability of thin-walled tubes, including composite overwrapping [19,20,21], foam or cellular filling [22,23,24,25,26], multi-cellular configurations [27,28], twin-tube systems [29,30,31,32], non-uniform wall-thickness design [12], bio-inspired tubes [33], and origami-inspired structures [34]. Fibre-based reinforcement, such as hybrid Al/composite tubes and graphene-modified composite crash boxes, has been shown to enhance peak force, absorbed energy, and specific energy absorption [19,21]. Internal filling strategies, including auxetic infills, foam-filled composite tubes, and plate-based metamaterial fillers, can further improve energy absorption, specific energy absorption, and crushing force efficiency while maintaining controlled peak forces [23,26,35]. Hierarchical and partitioned multi-cell designs also contribute to more stable crushing responses and enhanced energy dissipation [27,28]. These studies demonstrate that hybrid structural design and geometric modification can effectively regulate collapse modes and energy absorption. However, the specific role of bio-inspired perforation topology in controlling the interaction between a perforated outer tube and an inner conventional tube remains insufficiently understood.

Previous twin-tube and double-tube studies show that combining two structural components can increase axial load capacity, ductility, or energy absorption by constraining and redistributing deformation [29,30,31,32,36,37]. This provides the mechanical basis for the present inner–outer tube architecture. The study then asks how the outer-tube perforation pattern changes the collapse of the inner tube and how this affects peak force, mean crushing force, collapse stability, and energy absorption. These effects are assessed by comparing each hybrid tube with its corresponding inner and outer components.

Auxetic metamaterials, which exhibit a negative Poisson’s ratio, provide an effective route for tailoring mechanical response through structural topology. Unlike conventional materials, auxetic systems can undergo lateral contraction under compression and transverse expansion under tension, which can improve deformation stability and delay local failure. These features have been associated with enhanced compressive resistance [38], shear performance [39], energy absorption [40], fracture toughness [41], and acoustic attenuation [42]. Importantly, the mechanical response of auxetic metamaterials is strongly governed by their geometry. Zhou et al. [43] showed that the stiffness and auxeticity of a crystal-inspired auxetic metamaterial can be tuned through geometric parameters. Hierarchical auxetic honeycombs have also been shown to enhance stiffness and auxetic characteristics through geometric design [44]. In tubular structures, auxetic and other geometry-modified designs have been shown to modify deformation modes, reduce peak forces, promote smoother crushing, and improve specific energy absorption [36,45,46]. Similar geometry-driven mechanisms have also been reported in recent bio-inspired and additively manufactured metamaterials, where cellular topology and structural regularisation strongly affected compressive strength and failure behaviour [2]. These studies highlight the potential of auxetic tubular metamaterials but also indicate that the influence of bio-inspired outer-tube perforation geometry on hybrid tube deformation and energy dissipation remains insufficiently explored. In axial compression, the response of an auxetic-inspired tube depends on its ligament arrangement, tubular curvature, and interaction with the inner support tube. These factors determine whether deformation remains spatially distributed or becomes concentrated in premature local or global buckling.

Despite these advances, the geometry-dependent behaviour of auxetic–conventional hybrid tubular metamaterials has not yet been fully clarified. Earlier studies have demonstrated the feasibility of combining an auxetic outer tube with a conventional inner tube [36,37], while modified arrow-head and re-entrant auxetic structures have shown that ligament-level geometric design can improve energy absorption and compressive stability [47,48]. However, the influence of outer-tube perforation topology on the mechanical response of auxetic–conventional hybrid tubes remains unclear, particularly in terms of local buckling, load transfer, deformation stability, and energy partitioning between the inner and outer tubes. From a biomimetic design perspective, it is also necessary to establish how biological cellular and tubular features can be abstracted into regular engineering perforation patterns for lightweight tubular energy absorbers.

Regarding the materials used in previous auxetic–conventional hybrid tubes, Logakannan et al. [37] fabricated both the conventional inner tube and the perforated auxetic outer tube from AA6060-T5. Although the outer tube altered the deformation mode of the inner tube, its early failure resulted in the SEA of the hybrid tube being 38% lower than that of the conventional tube at a displacement of 50 mm. Huo et al. [36] subsequently combined an extruded AA6063-T5 inner tube with perforated 304 or 316 L stainless-steel outer tubes and showed that increasing the strength of the outer-tube material improved the load-carrying capacity and EA of the hybrid tube.

Motivated by this gap, the present study investigates bio-inspired auxetic–conventional hybrid tubular metamaterials consisting of a perforated stainless-steel outer tube and an aluminium inner tube. Four representative outer-tube perforation topologies, namely oval, circular, rotating-square, and re-entrant patterns, are investigated to examine the effects of pore geometry and ligament arrangement on collapse mode, load transfer, deformation stability, and energy absorption. The circular pattern was included as a conventional rounded-pore reference, while the oval, rotating-square, and re-entrant patterns were selected to examine pore elongation and ligament continuity, rotating-unit deformation, and re-entrant deformation, respectively. The biological-to-engineering translation is described in Section 2.1. Experimental axial compression tests are first conducted to compare the deformation modes, force–displacement responses, and energy absorption characteristics of single and hybrid tubes. A finite element model is then validated against the experimental results and used to further examine the effects of oval-hole aspect ratio and inner-tube wall thickness. The results provide a clearer understanding of how perforation topology and key geometric parameters govern the mechanical response of bio-inspired hybrid tubular energy absorbers, offering guidance for the design of lightweight protective and energy-absorbing structures.

The present study provides a controlled comparison of perforated outer-tube topologies within a modular hybrid system, rather than proposing the double-tube arrangement itself as a new concept. In contrast to previous twin-tube and double-tube studies that primarily examined the overall benefits of the combined arrangement, infill strategies, or global load capacity [29,30,31,32], four perforated outer-tube topologies were compared on the basis of approximately equal remaining CAD material volume, together with their corresponding inner and outer single components. This controlled comparison allows topology-dependent changes in collapse mode, peak force, mean crushing force, crushing force efficiency, energy absorption, and specific energy absorption to be distinguished from the general effect of adding a second tube. The subsequent Oval-Hybrid sensitivity study further treats pore elongation and inner-tube support stiffness as separate design variables. Thus, the differentiated value of this work is a comparative framework for relating outer-tube perforation topology and inner support to deformation regulation and crashworthiness trade-offs.

## 2. Materials and Methods

### 2.1. Bio-Inspired Design and Specimen Preparation

In this study, the inner tubes were extruded from 6063 aluminium alloy and underwent T5 heat treatment, while the outer tubes were made of 304 stainless steel and patterned with different perforation topologies. Both types of tubes were supplied by Xinghua Zhongsheng Metal Products Factory (Xinghua, Jiangsu, China). The aluminium and steel tubes were assembled without mechanical or chemical bonding, with a small clearance maintained between the inner and outer tubes. The aluminium inner tube and perforated outer tubes were also tested individually and compared with the corresponding hybrid tubes.

**Figure 2:**
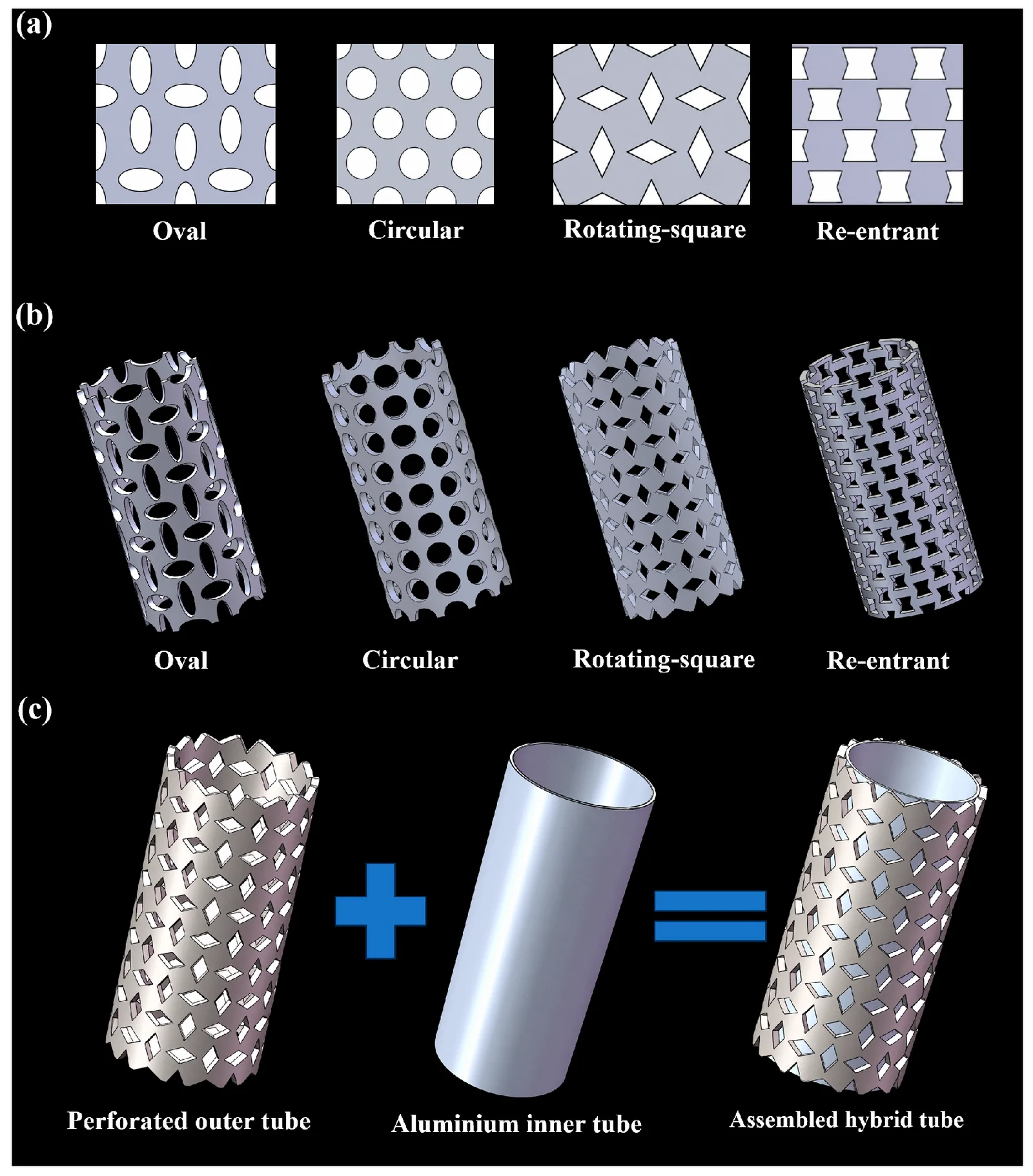
Engineering design route of the perforated outer tubes and the assembled auxetic–conventional hybrid tube: (**a**) representative two-dimensional perforation patterns, including oval, circular, rotating-square, and re-entrant patterns; (**b**) implementation of these patterns into three-dimensional perforated stainless-steel outer tubes; and (**c**) representative assembly of a perforated outer tube with an aluminium inner tube to form the hybrid tube.

Figure 2 illustrates the design route of the perforated outer tubes and the assembled auxetic–conventional hybrid tube. Based on the porous and cellular biological architectures summarised in Figure 1, the design strategy focused on functional principles including controlled porosity, pore-and-ligament continuity, shell-like material distribution, cellular load transfer, and core-supported tubular stability [4,5,6,7,8,9,10]. These principles were translated into an engineering design problem: selecting representative perforation topologies that can regulate local deformation, collapse stability, and energy absorption in hybrid tubular structures.

The distributed pore-and-ligament features of bony armour and the ordered porous walls of diatom frustules inspired the perforated outer tube [4,5]. The interconnected skeletal network of the glass sponge highlighted ligament connectivity and load transfer, while the hollow cellular structure of straw stems informed the hybrid configuration comprising a perforated outer tube and an inner support tube [6,7,8,9]. Within this framework, the circular pattern served as a conventional rounded-pore reference. The oval, rotating-square, and re-entrant patterns were selected to examine pore elongation and ligament continuity, rotating-unit deformation, and re-entrant deformation, respectively [36,37,38,39,40,41,42,43,44,45,46,47,48].

After the perforated outer tubes were fabricated, the hybrid tube was formed by combining each perforated stainless-steel outer tube with an aluminium inner tube. The perforated outer-tube geometries were modelled using SolidWorks 2023 (Dassault Systèmes SolidWorks Corp., Waltham, MA, USA). In this configuration, the perforated outer tube provided the topology-dependent deformation response, whereas the inner tube acted as a conventional support component. This design concept is consistent with the core-supported tubular strategy observed in natural cellular tubes, where the interaction between an outer shell and an internal support can improve resistance to local buckling, regulate collapse mode, and enhance energy absorption [9]. All perforations were fabricated by laser cutting. The single outer tubes were denoted as Oval-Single, Circle-Single, Square-Single, and Re-entrant-Single, corresponding to the oval, circular, rotating-square, and re-entrant patterns, respectively. The corresponding hybrid tubes were denoted as Oval-Hybrid, Circle-Hybrid, Square-Hybrid, and Re-entrant-Hybrid.

The dimensions of all specimens are listed in Table 1a. The values in Table 1a represent the measured dimensions of the as-fabricated specimens. The four perforated 304 stainless-steel outer tubes were not designed with an identical outer diameter or wall thickness. Instead, the wall thickness and consequently, the outer diameter, was adjusted according to the perforation topology so that the remaining CAD solid volumes and nominal outer-tube masses were approximately equal. The remaining CAD solid volumes were 22,795.32, 22,784.78, 22,803, and 22,777 mm^3^ for the Oval, Circle, Rotating-square, and Re-entrant outer tubes, respectively. The maximum difference was 26 mm^3^, corresponding to 0.11% of the mean volume. Thus, the comparison basis for the four perforated outer tubes was approximately equal remaining CAD material volume and nominal mass, rather than equal outer diameter or equal measured mass.

**Figure 3:**
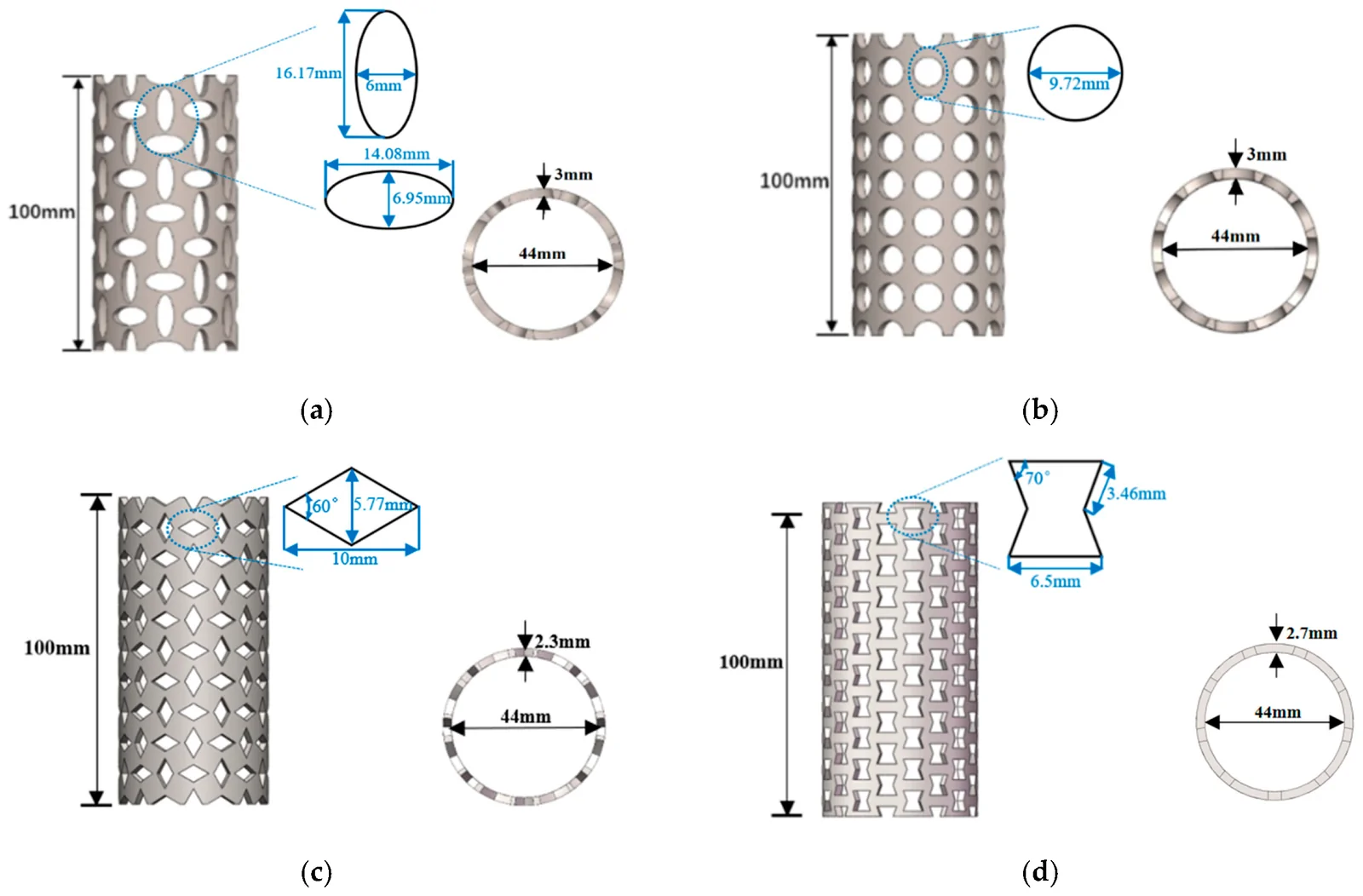
Geometric designs of perforated outer tubes: (**a**) Oval-Single; (**b**) Circle-Single; (**c**) Square-Single; (**d**) Re-entrant-Single.

In this study, porosity is defined as the perforation-induced material-removal ratio and excludes the central hollow bore. To maintain a consistent calculation basis, both the unperforated reference volume and the remaining perforated volume were obtained from the same nominal CAD geometry for each topology; the measured dimensions in Table 1a were not substituted into this calculation:

(1) $$V_{0,k}=\frac{\pi }{4}\times ({D_{o,k}}^{2}-{D_{i,k}}^{2})\times L_{k}$$

(2) $$P_{k}=\frac{V_{0,k}-V_{p,k}}{V_{0,k}}\times 100\%$$

where *V*_0,_*_k_* and *P_k_* are the unperforated CAD reference volume and material-removal porosity for topology *k*, respectively; *D*_o,_*_k_*, *D*_i,_*_k_*, and *L_k_* are the nominal outer diameter, inner diameter, and length of the unperforated CAD precursor for topology *k*, respectively; and *V*_p,_*_k_* is the remaining solid volume of the corresponding perforated CAD model. The nominal precursor dimensions (*D*_o_/*D*_i_/*t*/*L*, in mm) were 50.0/44.0/3.0/100 for the Oval and Circle tubes, 48.6/44.0/2.3/100 for the Rotating-square tube, and 49.4/44.0/2.7/100 for the Re-entrant tube. The corresponding unperforated CAD reference volumes were 44,296.46, 44,296.46, 33,454.82, and 39,612.34 mm^3^, respectively, giving material-removal porosities of 48.54%, 48.56%, 31.84%, and 42.50%. The measured masses of specimens S1–S3 and their mean ± sample standard deviation are reported in Table 1b. For each hybrid specimen, the reported mass includes both the perforated steel outer tube and the aluminium inner tube. As shown in Figure 3, the perforation layouts were arranged periodically along both the axial and circumferential directions, and the detailed unit dimensions are provided in Figure 3.

### 2.2. Material Characterisation

**Figure 4:**
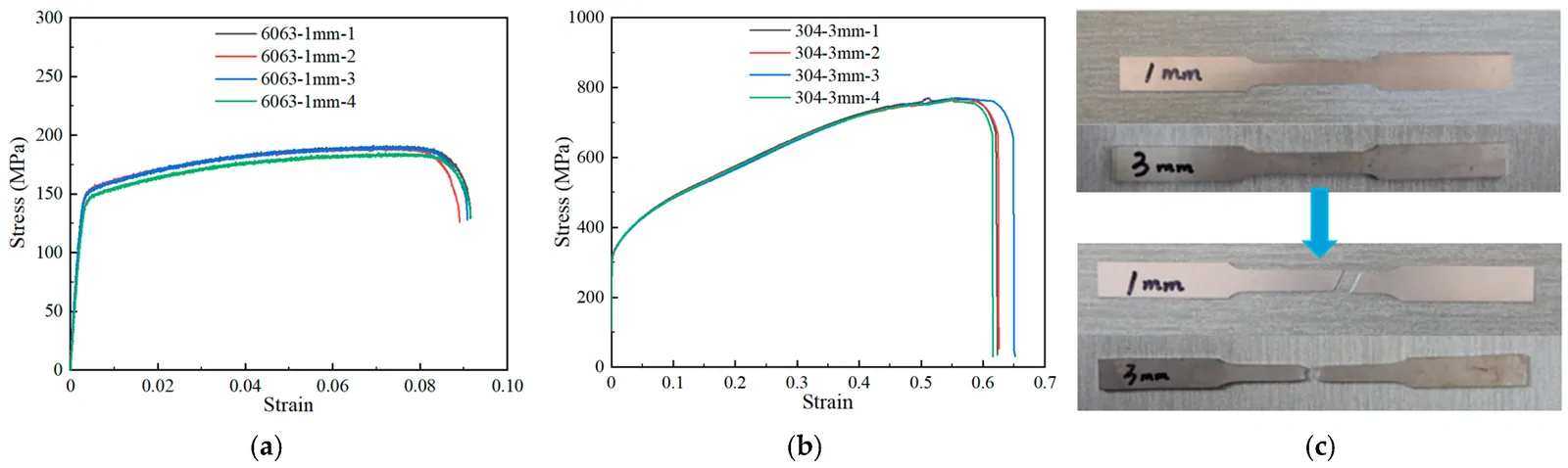
Tensile characterisation of tube-wall materials: (**a**) stress–strain curves of aluminium alloy 6063-T5 coupons, (**b**) stress–strain curves of stainless steel 304 coupons, and (**c**) dog-bone specimens before and after tensile testing.

The mechanical properties of the tube-wall materials were determined by standard uniaxial tensile tests on dog-bone coupons in accordance with ASTM E8/E8M [49]. The tensile tests were conducted using an electro-hydraulic servo testing machine at a constant crosshead speed of 2.00 mm/min. Coupons cut from aluminium alloy 6063-T5 and 304 stainless-steel tubes, with nominal thicknesses of 1 mm and 3 mm, respectively, were tested and denoted as 6063-1 mm and 304-3 mm. Four replicate tests were performed for each material to obtain representative mechanical properties for both experimental interpretation and finite element modelling. Figure 4a,b show the engineering stress–strain curves obtained from the tensile tests, while Figure 4c presents the dog-bone specimens before and after testing. The measured material properties are summarised in Table 2.

### 2.3. Uniaxial Compressive Tests of Tubes

Quasi-static uniaxial compression tests were performed using a pressure testing machine with a load capacity of 1000 kN. All single and hybrid tube specimens were placed between two rigid compression platens and compressed axially at a constant crosshead speed of 3 mm/min. The tests were terminated at an axial displacement of 15 mm, corresponding to approximately 15% of the initial tube length. Based on the experimental observations, this common displacement range captured the principal early-stage crushing response and was adopted for consistent comparison among the tube configurations. During testing, the force–displacement response was recorded by the testing machine, and the deformation history of each specimen was captured using a Canon EOS R10 digital camera (Canon Inc., Tokyo, Japan). The obtained force–displacement curves were subsequently used to evaluate the crashworthiness and energy-absorption metrics of the single and hybrid tubes. For each configuration, three independent specimens were tested. The mean and sample standard deviation of *P*_initial_, *F*_max_, MCF, CFE, EA, and SEA were calculated from the three replicate tests and are reported as the mean ± standard deviation.

## 3. Results and Discussion

### 3.1. Deformation Modes of Single Tubes

**Figure 5:**
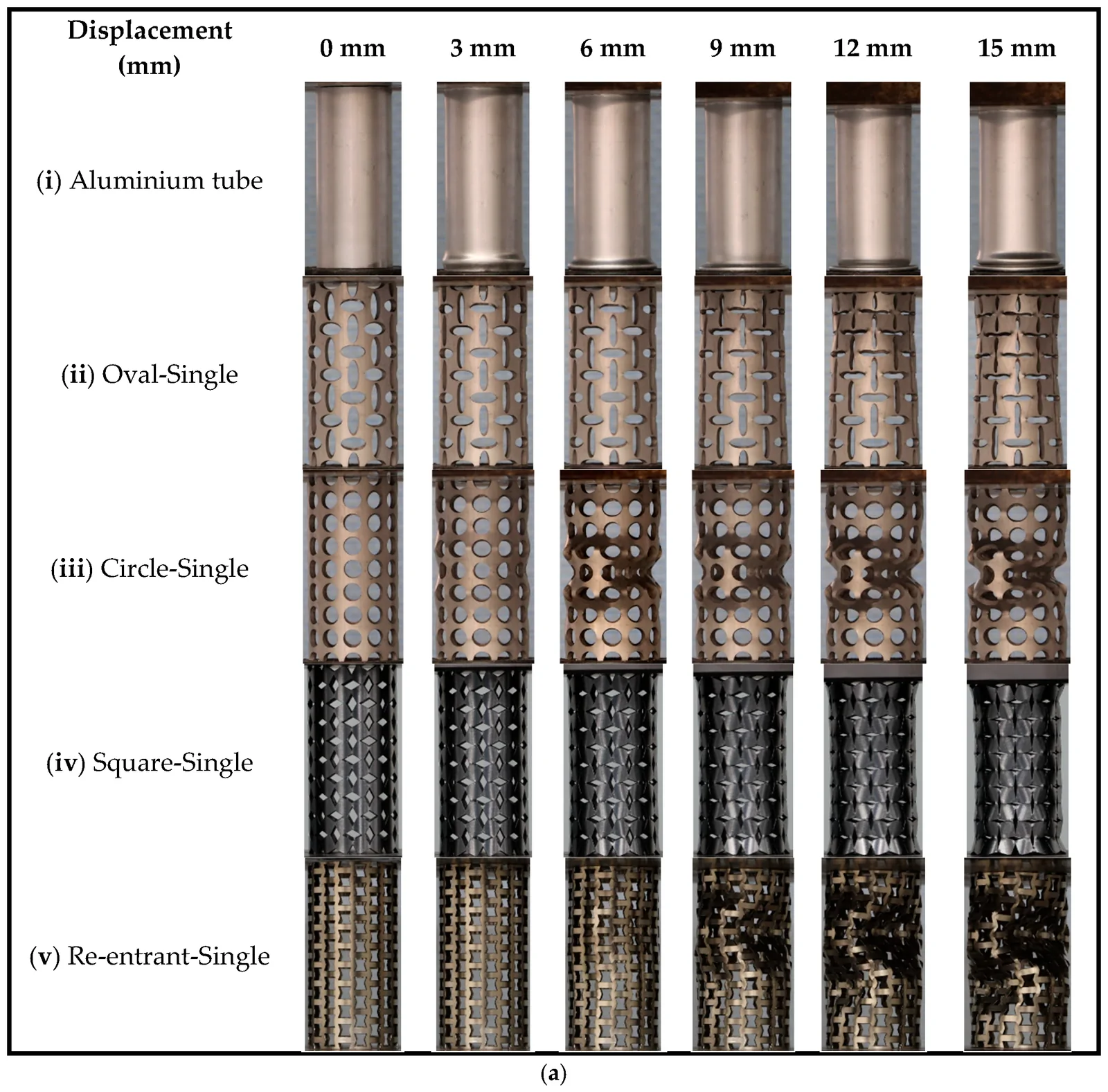

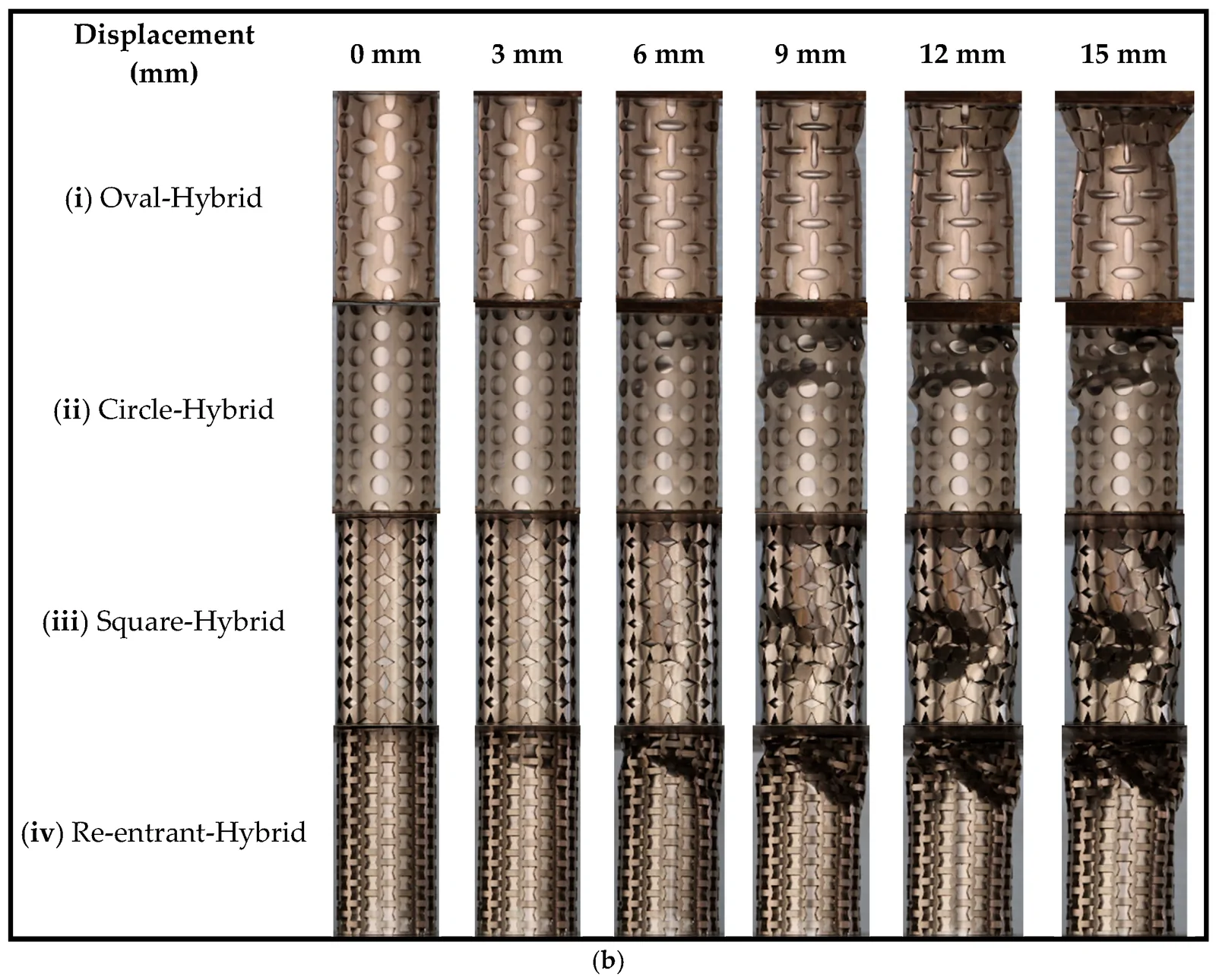
Experimental deformation modes of single and hybrid tubes under axial compression: (**a**) single tubes; and (**b**) hybrid tubes.

The replicate tests showed consistent deformation processes for each configuration. Therefore, representative deformation modes of the single and hybrid tubes at selected axial displacements are shown in Figure 5.

**Figure 6:**
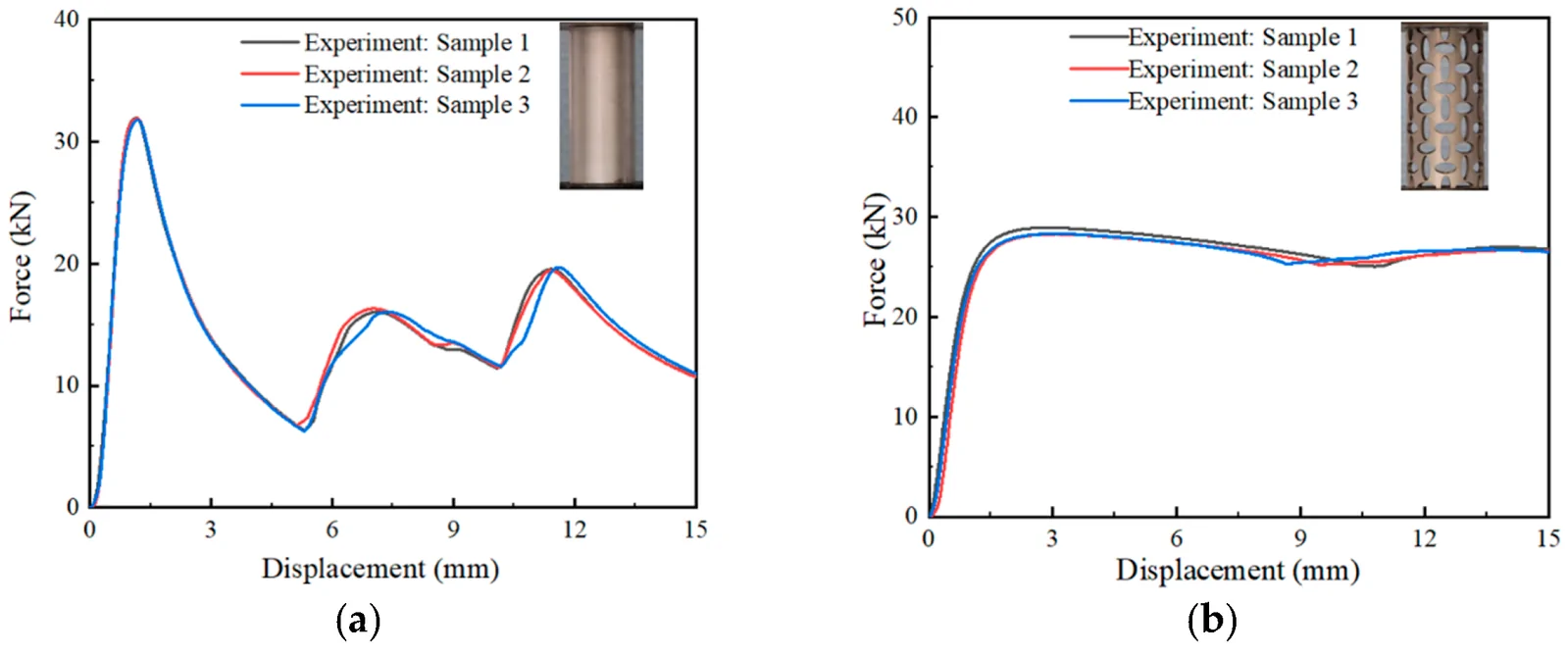

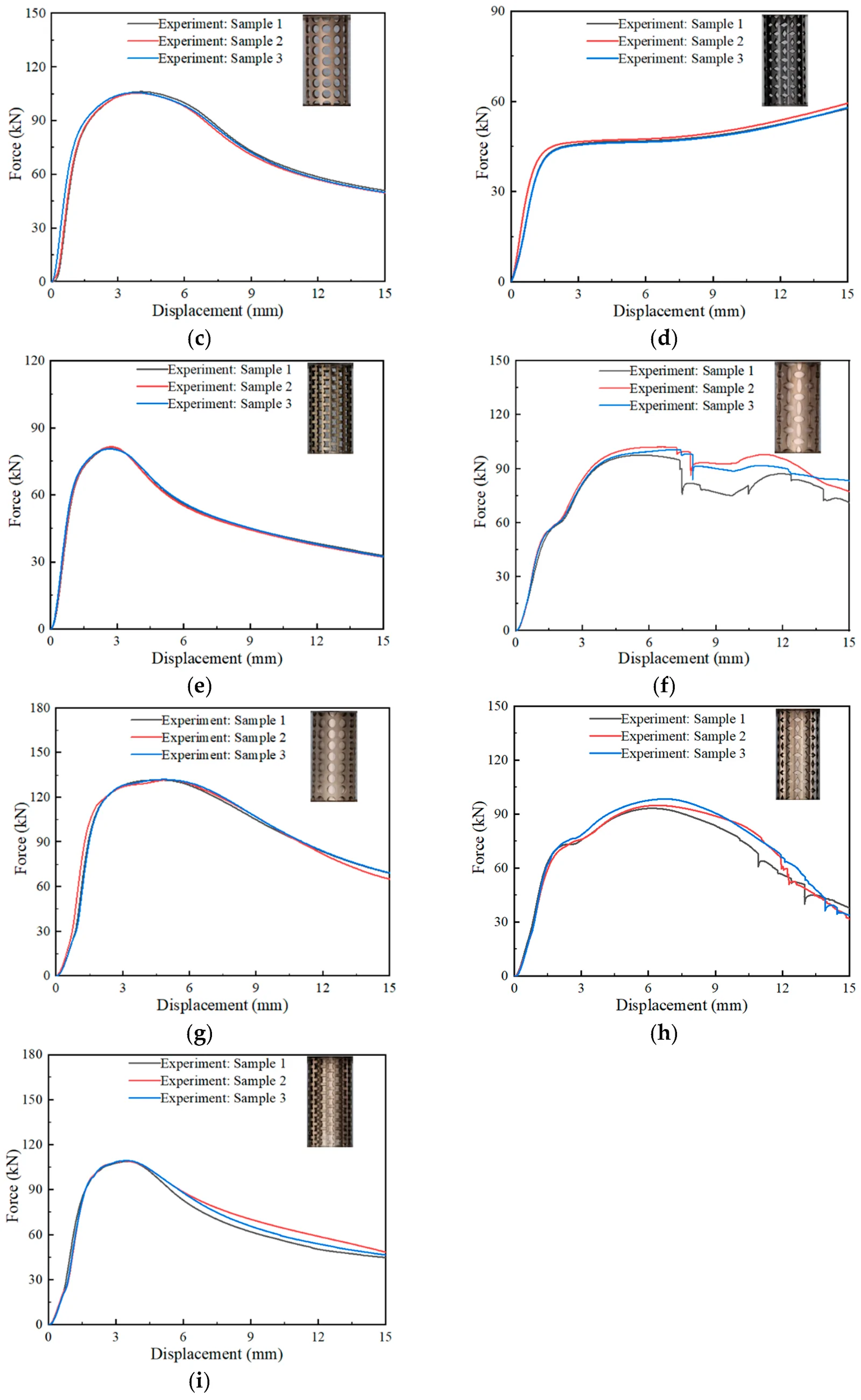
Force–displacement curves of single and hybrid tubes: (**a**) aluminium tube, (**b**) Oval-Single, (**c**) Circle-Single, (**d**) Square-Single, (**e**) Re-entrant-Single, (**f**) Oval-Hybrid, (**g**) Circle-Hybrid, (**h**) Square-Hybrid, and (**i**) Re-entrant-Hybrid.

The conventional aluminium tube exhibited a typical ring-mode collapse under axial compression, characterised by the progressive formation of circumferential lobes (Figure 5a(i)). The first lobe appeared at approximately 6 mm displacement, and three distinct force fluctuations were observed in the force–displacement response (Figure 6a). The initial peak force was approximately 31.90 kN at 1.20 mm displacement, followed by subsequent peaks of approximately 16.00 kN and 19.50 kN at around 5.20 mm and 15.00 mm, respectively. This progressive folding behaviour provided a stable deformation mode for energy dissipation.

As shown in Figure 5a(ii,iv) and Figure 6b,d, the Oval-Single and Square-Single tubes exhibited more stable deformation and force–displacement responses than the Circle-Single and Re-entrant-Single tubes. The Oval-Single tube reached a maximum force of approximately 28.51 kN, whereas the Square-Single tube exhibited an initial peak force of approximately 46.62 kN and a maximum force of 58.32 kN. Both tubes showed transverse and radial contraction associated with the deformation characteristics of their respective perforation topologies. The Oval-Single tube displayed localised deformation in the upper region, whereas the deformation of the Square-Single tube was more evenly distributed along the tube height. Despite this difference, both tubes exhibited a similar force–displacement trend, with a sharp initial force increase followed by stabilisation into a plateau region. For the Oval-Single tube, the mean crushing force was 25.87 kN, while the Square-Single tube showed a similar response but with more pronounced outward flaring.

The Circle-Single tube exhibited a deformation mode dominated by localised buckling and outward bulging, primarily concentrated in the central region and near the tube ends. The force–displacement curve in Figure 6c shows a peak force of approximately 105.88 kN at the onset of local instability. As the displacement increased, the force gradually decreased, indicating the development of inward deformation and structural instability associated with the circular perforation topology.

The Re-entrant-Single tube also showed localised buckling and outward bulging, with deformation mainly concentrated in the central and upper regions (Figure 5a(v)). The force reached a peak of approximately 81.09 kN (Figure 6e), marking the onset of localised plastic deformation. With increasing displacement, localised buckling became more prominent, leading to a gradual reduction in force as the buckling propagated along the perforation pattern. Although re-entrant geometries are commonly associated with auxetic behaviour, the present tubular configuration showed an instability-dominated response under axial compression.

In summary, the single-tube results demonstrate that the bio-inspired perforation topology strongly affected the deformation mode and load-bearing response. The Oval-Single and Square-Single tubes showed more stable contraction-related deformation, whereas the Circle-Single and Re-entrant-Single tubes were more prone to localised buckling, inward deformation, and post-peak force reduction. This indicates that the regularised pore geometry of the perforated outer tube plays an important role in controlling local instability and progressive deformation under axial compression.

### 3.2. Deformation Modes of Hybrid Tubes

The hybrid tubes exhibited more complex deformation responses than the corresponding single tubes because of the interaction between the perforated stainless-steel outer tube and the aluminium inner tube. As shown in Figure 5b(i–iv) and Figure 6f–i, the outer-tube perforation topology strongly influenced the collapse mode, force evolution, and load transfer between the two components.

For the Oval-Hybrid tube, the force initially increased sharply, reflecting the combined elastic response of the aluminium inner tube and the stainless-steel outer tube (Figure 6f). At approximately 2 mm displacement, the slope of the force–displacement curve slightly decreased, which can be attributed to the onset of localised yielding and stress redistribution within the hybrid structure. As compression progressed, the force gradually stabilised into a plateau region, accompanied by localised buckling and transverse contraction in the upper region of the tube (Figure 5b(i)). The oval perforation topology promoted a controlled contraction-dominated deformation mode and delayed severe global instability. The peak force reached approximately 100.05 kN at a displacement of 6.56 mm. A sudden force drop occurred at approximately 7.5 mm displacement, which was associated with fracture around the perforated region of the outer tube. By 15 mm displacement, localised buckling and outward bulging became more evident, while the hybrid tube still maintained a relatively stable load-bearing response.

The Circle-Hybrid tube showed a higher peak force than the Oval-Hybrid tube but a less stable post-peak response. As shown in Figure 6g, the force increased rapidly during the initial stage and reached a peak of approximately 131.79 kN at 5.5 mm displacement. After this peak, the force began to decrease, mainly due to localised buckling in the central region of the tube (Figure 5b(ii)). At larger displacements, outward flaring near the upper region and local instability around the circular perforations became more pronounced. This response indicates that the circular perforation topology provided high initial load resistance but was more susceptible to localised collapse after peak loading.

The Square-Hybrid tube exhibited diamond-shaped localised buckling and an asymmetrical folding mode (Figure 5b(iii)). During the initial elastic stage, the force increased almost linearly. At approximately 2.5 mm displacement, the slope of the force–displacement curve decreased, which is attributed to localised yielding and internal stress redistribution before the development of structural buckling. The force reached a peak of approximately 95.52 kN at around 6 mm displacement (Figure 6h). With further compression, irregular and asymmetrical buckling became more evident, indicating that the interaction between the rotating-square outer tube and the aluminium inner tube produced a more complex deformation response than that of the corresponding single tube.

The Re-entrant-Hybrid tube showed localised buckling concentrated mainly in the upper section of the structure (Figure 5b(iv)). The force–displacement curve exhibited an initial sharp increase and reached a peak force of approximately 109.10 kN at 4.5 mm displacement (Figure 6i). After the peak, the force decreased because of the development of localised buckling and inward folding near the upper edge, which reduced the load-bearing capacity. By 15 mm displacement, the deformation had evolved into an asymmetrical buckling mode, while the lower section of the tube remained comparatively less deformed. This behaviour indicates that the re-entrant perforation topology did not produce a uniformly distributed contraction mode in the present tubular configuration.

Overall, outer-tube topology determined how the aluminium inner tube collapsed and retained load. The Oval-Hybrid tube maintained a comparatively distributed contraction-dominated mode and achieved an MCF of 81.89 ± 3.88 kN, a CFE of 81.82 ± 2.03%, and an SEA of 5.76 ± 0.25 kJ/kg. In contrast, the Re-entrant-Hybrid tube developed localised inward folding and asymmetric buckling near its upper end. Despite reaching an initial peak force of 109.10 kN, it achieved an MCF of 70.59 ± 2.25 kN, a CFE of 64.70 ± 2.01%, and an SEA of 5.05 ± 0.13 kJ/kg. Its localised and asymmetric collapse caused the pronounced post-peak load reduction and limited its energy-absorption efficiency.

### 3.3. Crashworthiness and Energy-Absorption Metrics

The initial peak force (*P*_initial_), maximum force (*F*_max_), mean crushing force (MCF), crushing force efficiency (CFE), energy absorption (EA), and specific energy absorption (SEA) were used to quantitatively evaluate the crashworthiness performance of the single and hybrid tubes. The corresponding quantitative results are summarised in Table 3. *P*_initial_ denotes the first peak force during axial compression, whereas *F*_max_ represents the maximum force recorded within the prescribed 0–15 mm compression range. These force-related indicators are important for evaluating the initial crushing resistance and the peak load transmitted by the structure.

**Table 3:** Crashworthiness metrics of single and hybrid tubes. Values are reported as the mean ± standard deviation (*n* = 3).

Type	*P*_initial_ (kN)	*F*_max_ (kN)	MCF (kN)	CFE (%)	EA (kJ)	SEA (kJ/kg)
Aluminium tube	31.87 ± 0.08	31.87 ± 0.08	14.59 ± 0.05	45.80 ± 0.11	0.219 ± 0.001	6.03 ± 0.13
Oval-Single	28.51 ± 0.37	28.51 ± 0.37	25.87 ± 0.30	90.75 ± 0.40	0.388 ± 0.005	2.22 ± 0.05
Circle-Single	105.88 ± 0.41	105.88 ± 0.41	75.79 ± 0.80	71.58 ± 0.63	1.137 ± 0.012	6.40 ± 0.12
Square-Single	46.62 ± 0.42	58.32 ± 0.94	46.93 ± 0.88	80.48 ± 0.49	0.704 ± 0.013	4.20 ± 0.11
Re-entrant-Single	81.09 ± 0.45	81.09 ± 0.45	50.75 ± 0.32	62.59 ± 0.74	0.761 ± 0.005	4.42 ± 0.06
Oval-Hybrid	100.05 ± 2.31	100.05 ± 2.31	81.89 ± 3.88	81.82 ± 2.03	1.229 ± 0.058	5.76 ± 0.25
Circle-Hybrid	131.79 ± 0.24	131.79 ± 0.24	99.02 ± 0.39	75.14 ± 0.24	1.485 ± 0.006	6.95 ± 0.04
Square-Hybrid	75.04 ± 1.74	95.52 ± 2.65	70.74 ± 2.07	74.06 ± 0.38	1.061 ± 0.031	5.24 ± 0.17
Re-entrant-Hybrid	109.10 ± 0.28	109.10 ± 0.28	70.59 ± 2.25	64.70 ± 2.01	1.059 ± 0.034	5.05 ± 0.13

Energy absorption (EA) quantifies the energy dissipated within the prescribed compression range and is calculated as the area under the force–displacement curve:

(3) $$EA=\int_{0}^{d}F(x)dx$$

where F and x represent the compressive force and displacement, respectively.

Specific energy absorption (SEA), defined as the energy absorbed per unit mass, is calculated as:

(4) SEA=EA/m

where *m* is the total specimen mass. For a hybrid tube, the mass includes both the perforated stainless-steel outer tube and the aluminium inner tube.

Mean crushing force (MCF) represents the average force sustained during compression and is obtained by:

(5) MCF=EA/d

where d is the compression displacement.

Crushing force efficiency (CFE), defined as the ratio of MCF to *F*_max_, evaluates the stability of the crushing response relative to the peak load:

(6) $$CFE=MCF/F_{max}\times 100\%$$

**Figure 7:**
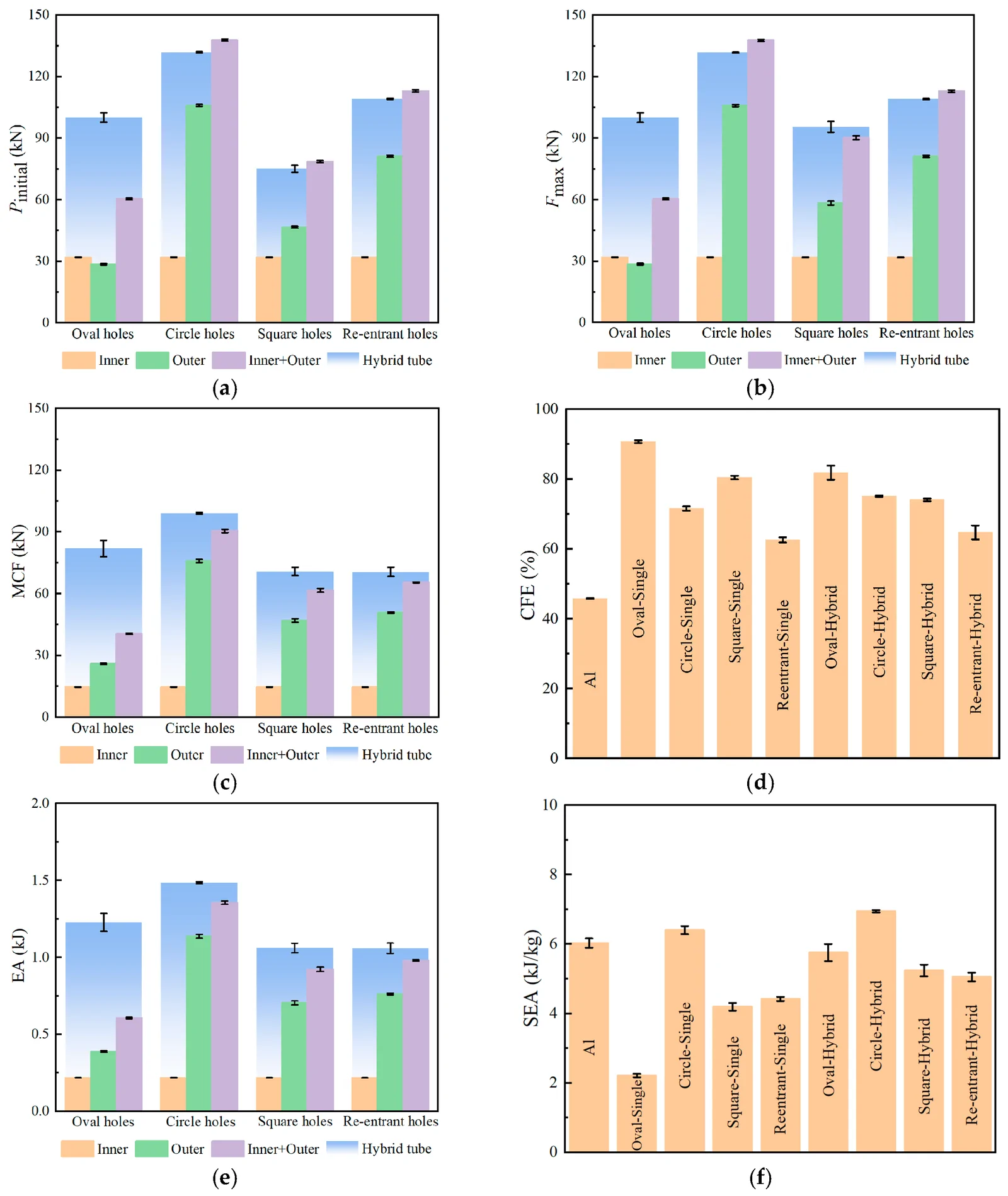
Comparison of crashworthiness metrics for single and hybrid tubes: (**a**) *P*_initial_, (**b**) *F*_max_, (**c**) MCF, (**d**) CFE, (**e**) EA, and (**f**) SEA. Bars represent mean values, and error bars indicate ± one standard deviation based on three independent tests (*n* = 3).

As shown in Figure 7a,b, the single tubes exhibited distinct topology-dependent force responses. For the Square-Single tube, *F*_max_ was higher than *P*_initial_, whereas these two values were identical for the other single-tube configurations. Among the single tubes, the Circle-Single tube showed the highest *P*_initial_ and *F*_max_, while the Oval-Single tube showed the lowest values. For the hybrid tubes, the Circle-Hybrid tube exhibited the highest *P*_initial_ and *F*_max_, followed by the Re-entrant-Hybrid, Oval-Hybrid, and Square-Hybrid tubes. Compared with the combined values of its corresponding inner and outer single tubes, the Oval-Hybrid tube showed a 65.70% increase in both *P*_initial_ and *F*_max_, indicating a strong strengthening effect induced by the hybrid configuration. The Square-Hybrid tube also showed a 5.91% increase in *F*_max_. In contrast, the *P*_initial_ values of the Circle-Hybrid, Square-Hybrid, and Re-entrant-Hybrid tubes were slightly lower than the combined values of their corresponding single components, with reductions of 4.33%, 4.40%, and 3.42%, respectively.

As shown in Figure 7c, all hybrid tubes showed higher MCF values than the sum of their corresponding single components, with increases of 102.40%, 9.56%, 14.99%, and 8.03% for the oval, circular, rotating-square, and re-entrant configurations, respectively. This indicates that hybridisation improved the average load-bearing capacity during compression, particularly for the oval topology. The pronounced increase in the Oval-Hybrid tube suggests that the bio-inspired oval perforation pattern promoted contraction-dominated deformation of the outer tube, which enhanced its interaction with the aluminium inner tube and resulted in more effective load transfer and energy dissipation.

The CFE values are shown in Figure 7d. Quantitatively, the Oval-Hybrid tube maintained an MCF of 81.89 ± 3.88 kN relative to a maximum force of 100.05 ± 2.31 kN, resulting in the highest CFE among the hybrid configurations, at 81.82 ± 2.03%. This relatively high force-retention efficiency is consistent with its comparatively stable and distributed collapse mode. Although the Circle-Hybrid tube achieved the highest maximum force and MCF, at 131.79 ± 0.24 kN and 99.02 ± 0.39 kN, respectively, its CFE was 75.14 ± 0.24%, which was 6.68 percentage points lower than that of the Oval-Hybrid tube. This difference indicates that the high peak load of the Circle-Hybrid tube was not maintained proportionally over the prescribed compression displacement, consistent with the localised post-peak buckling observed experimentally. The Square-Hybrid and Re-entrant-Hybrid tubes exhibited CFE values of 74.06 ± 0.38% and 64.70 ± 2.01%, respectively. In particular, the Re-entrant-Hybrid tube reached a relatively high maximum force of 109.10 ± 0.28 kN but maintained an MCF of only 70.59 ± 2.25 kN, quantitatively reflecting the pronounced post-peak load reduction caused by its localised asymmetric collapse. Compared with their corresponding single outer tubes, the Circle-Hybrid and Re-entrant-Hybrid tubes showed relative CFE increases of 4.97% and 3.37%, respectively, whereas the CFE values of the Oval-Hybrid and Square-Hybrid tubes decreased by 9.84% and 7.98%, respectively. For the latter two configurations, this reduction occurred because the increase in maximum force was more pronounced than the increase in MCF. Nevertheless, all single and hybrid perforated tubes exhibited higher CFE values than the conventional aluminium tube, indicating more stable force distribution during axial crushing.

The EA values of the tubes are presented in Figure 7e. Because EA was calculated over the same compression displacement for all specimens, the trend in EA was consistent with that of MCF. The circular topology showed the highest EA among both the single and hybrid tubes, while the Oval-Hybrid tube ranked second among the four hybrid configurations.

The SEA values further demonstrate the topology-dependent effect of hybridisation on mass-normalised energy absorption (Figure 7f). The Circle-Hybrid tube achieved the highest mean SEA of 6.95 ± 0.04 kJ/kg, followed by the Oval-Hybrid tube at 5.76 ± 0.25 kJ/kg. Relative to their corresponding single tubes, the Circle-Hybrid and Oval-Hybrid tubes showed SEA increases of 8.59% and 159.46%, respectively. The Square-Hybrid and Re-entrant-Hybrid tubes achieved SEA values of 5.24 ± 0.17 and 5.05 ± 0.13 kJ/kg, corresponding to increases of 24.76% and 14.25%, respectively. Although the mean SEA of the Oval-Hybrid tube was 17.12% lower than that of the Circle-Hybrid tube, the Oval-Hybrid tube exhibited a 24.08% lower maximum force and a CFE that was 6.68 percentage points higher. Thus, the circular topology favoured higher mass-normalised energy absorption, whereas the oval topology provided a different trade-off between peak-force control and crushing efficiency. Among the hybrid configurations, only the Circle-Hybrid tube exceeded the mean SEA of the conventional aluminium tube. The engineering significance of the hybrid configuration therefore lies not only in maximising SEA, but also in enabling the outer-tube topology and inner-tube support to be adjusted independently to balance peak-force control, crushing stability, load transfer, energy absorption, and structural mass. The lower SEA values of the other hybrid tubes reflect the mass penalty associated with the stainless-steel outer tube.

Compared with fully 3D-printed cellular structures, the present hybrid-tube design provides a modular load-sharing mechanism. The perforated stainless-steel outer tube regulates local deformation and collapse mode, whereas the continuous aluminium inner tube provides axial support and redistributes the load. This interaction increased MCF by 8.03–102.40% relative to the summed values of the corresponding individual components and increased SEA by 8.59–159.46% relative to the corresponding perforated single tubes. The topology-dependent response also enables different performance priorities: the circular pattern maximised mass-normalised energy absorption, whereas the oval pattern provided a lower maximum force and higher CFE. In addition, the outer-tube perforation geometry and inner-tube properties can be adjusted independently, while the use of extruded and laser-cut metallic tubes provides a direct fabrication route without layer-interface or build-direction effects. Nevertheless, the additional inner tube introduces a mass penalty, and only the Circle-Hybrid tube exceeded the SEA of the conventional aluminium tube. The proposed design also has lower geometric freedom than fully 3D-printed cellular structures, which can realise complex three-dimensional, hierarchical, graded, and multifunctional architectures [50].

## 4. Finite Element Modelling

**Figure 8:**
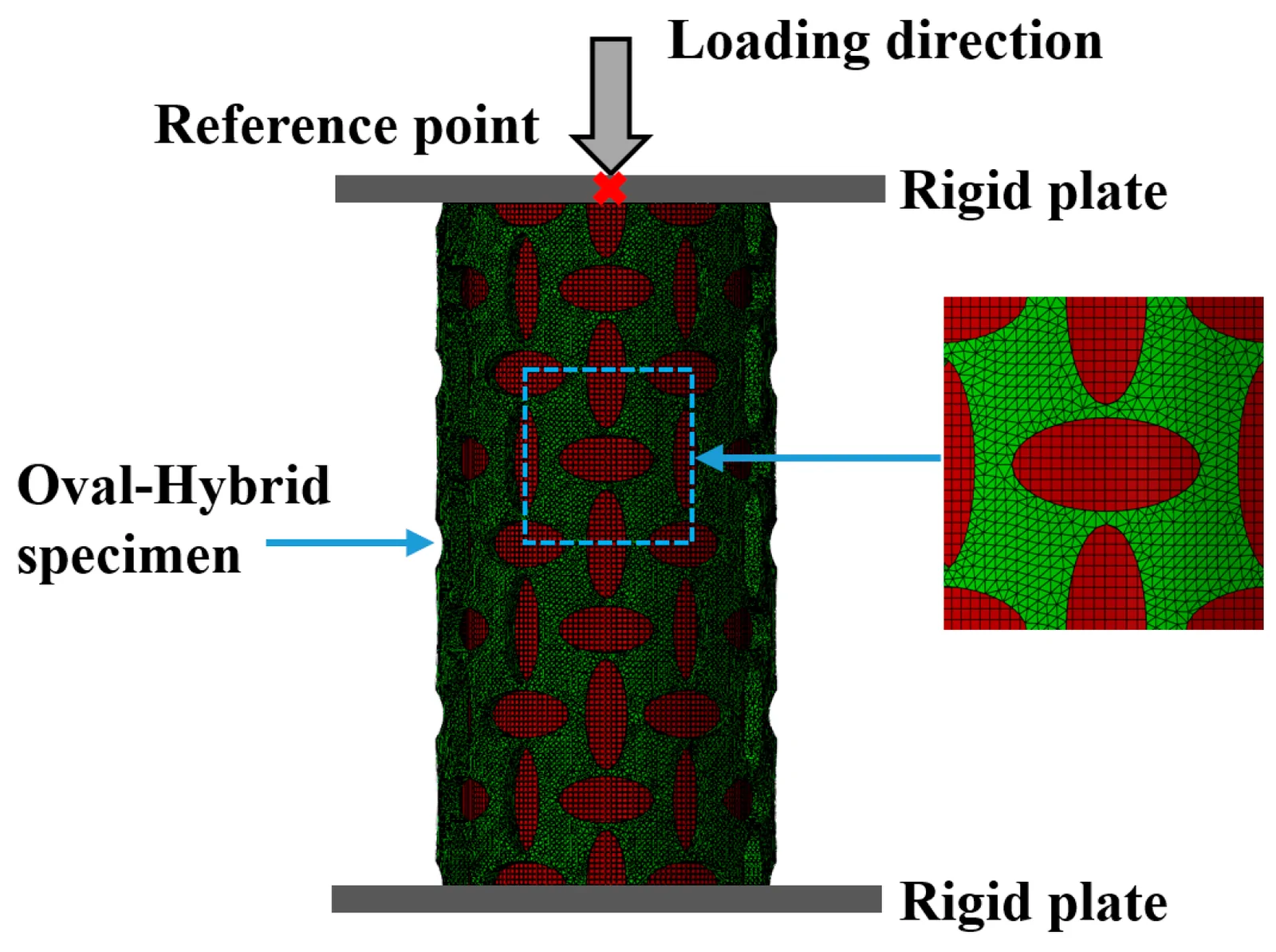
Finite element model of the Oval-Hybrid tube, showing the axial loading arrangement and an enlarged view of the mesh distribution around the perforation edges. The red cross indicates the reference point of the upper rigid plate.

A finite element model (FEM) of the Oval-Hybrid tube was developed using Abaqus/Explicit 2023 (Dassault Systèmes, Providence, RI, USA) to simulate its quasi-static uniaxial compression response, as shown in Figure 8. The explicit solver was adopted to capture large deformation, local buckling, and complex contact interactions during compression. The hybrid tube was placed between two rigid plates, with the bottom plate fully constrained and the top plate subjected to a prescribed 15 mm downward displacement through a reference point located at its centre. General contact was defined among the perforated outer tube, aluminium inner tube, and rigid plates, including self-contact of both deformable tube components. The tangential behaviour was modelled using a penalty friction formulation with a friction coefficient of 0.25, while hard contact was used in the normal direction.

The perforated stainless-steel outer tube was discretised using four-node linear tetrahedral solid elements (C3D4) with a nominal mesh size of 0.8 mm to accommodate the complex geometry around the perforation edges. The geometrically regular aluminium inner tube was discretised using eight-node linear brick elements with reduced integration and hourglass control (C3D8R), also with a nominal mesh size of 0.8 mm. Both formulations support finite-deformation elastic–plastic analysis in Abaqus/Explicit. The C3D4 formulation facilitated conforming meshing of the perforated outer tube, whereas C3D8R provided an efficient structured discretisation of the inner tube.

A five-level mesh-convergence study was conducted using nominal mesh sizes ranging from 2.0 to 0.6 mm. Relative to the 0.6 mm mesh, the selected 0.8 mm mesh differed by 1.61% in maximum force and 2.97% in energy absorption up to a displacement of 15 mm. The 0.8 mm mesh was therefore adopted for model validation and the subsequent parametric simulations. Further details of the mesh-convergence study are provided in Appendix A.1.

The tube materials were represented by isotropic linear elasticity followed by von Mises plasticity with isotropic hardening. Density, Young’s modulus, and Poisson’s ratio were assigned using the experimentally determined properties listed in Table 2. The engineering stress–strain curves obtained from the tensile tests were converted into true stress–true plastic strain data over their common pre-necking range and entered into Abaqus to define the plastic hardening response. To reduce computational cost, a prescribed displacement rate of 19.80 mm/min was used in the numerical simulations. An additional simulation performed at the experimental rate of 3.0 mm/min produced a closely comparable force–displacement response, while the ratio of kinetic energy to internal energy remained below 5%. These results confirmed that the adopted loading rate did not materially affect the predicted quasi-static response. Details of the loading-rate verification are provided in Appendix A.2.

## 5. Finite Element Validation

The Oval-Hybrid tube was selected for finite element validation and subsequent parametric analysis based on its combined crashworthiness performance and parameterisable pore geometry. Its maximum force was 24.08% lower than that of the Circle-Hybrid tube, while its CFE was the highest among the hybrid configurations and exceeded that of the Circle-Hybrid tube by 6.68 percentage points. Although its mean SEA of 5.76 ± 0.25 kJ/kg was 17.12% lower than that of the Circle-Hybrid tube, it showed the largest relative SEA improvement, approximately 159.46%, over its corresponding single tube. The oval-hole geometry also allowed its aspect ratio to be varied systematically to examine how pore elongation affects peak force, crushing stability, deformation mode, and energy absorption. The following numerical analysis therefore focuses on parameter effects within the Oval-Hybrid configuration, while comparisons among the four perforation topologies remain based on the experimental results.

**Figure 9:**
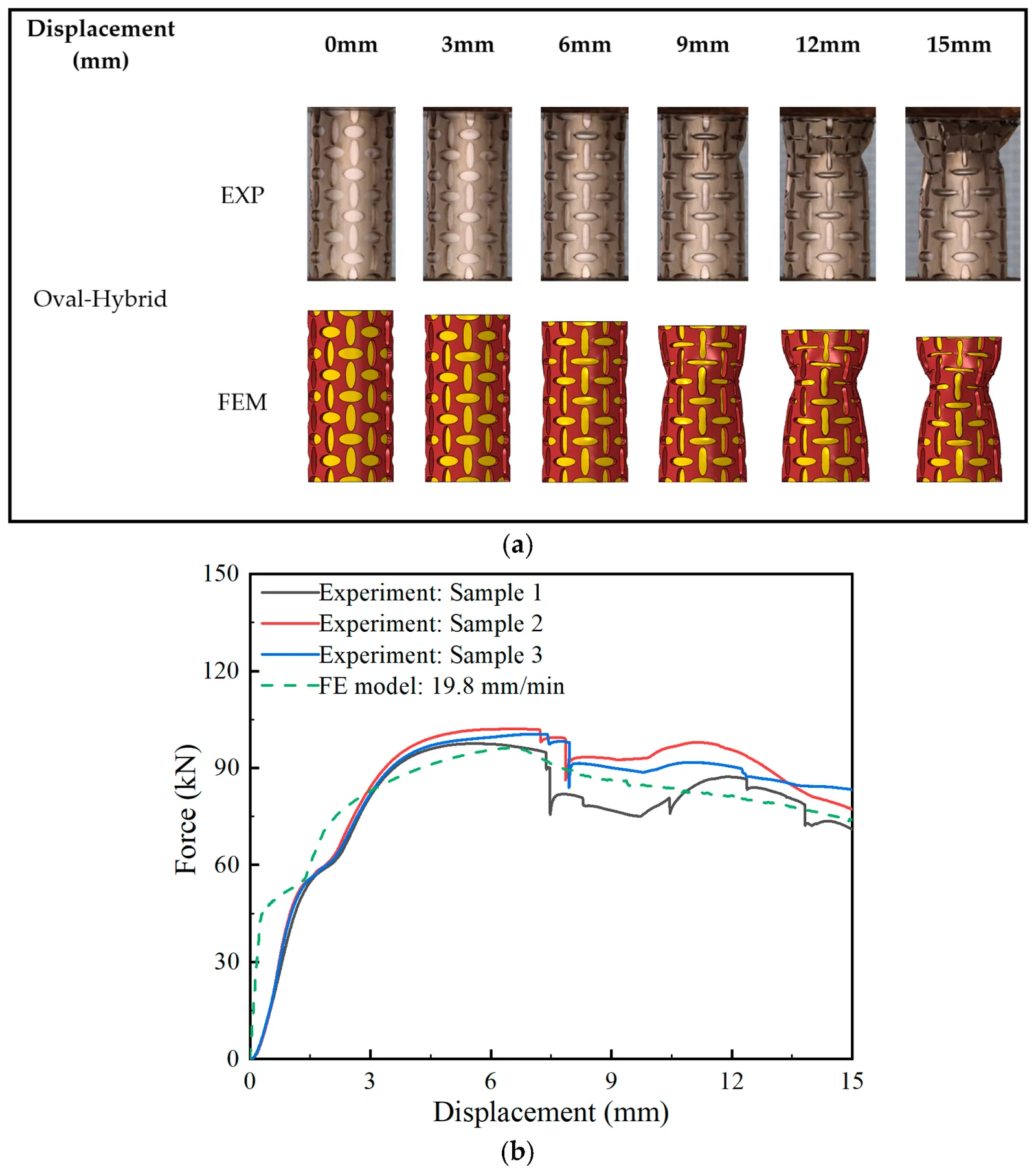
Finite element validation of the Oval-Hybrid tube: (**a**) comparison of the deformation sequences obtained from experimental Sample 1 and the finite element model at selected crushing displacements; and (**b**) comparison of the finite element prediction at 19.80 mm/min with the force–displacement curves obtained from all three experimental replicates.

The deformation mode and force–displacement response of the Oval-Hybrid tube obtained from the finite element simulation were compared with the experimental results, as shown in Figure 9. The experimental deformation sequence presented in Figure 9a corresponds to Oval-Hybrid Sample 1. Overall, the FEM results captured the main deformation characteristics observed in the experiment, including localised bulging, radial contraction, and progressive deformation of the perforated outer tube. A discrepancy was observed in the initiation position of contraction. In the experiment, contraction initiated at approximately one-third of the specimen height from the top, whereas the simulation predicted a more symmetric deformation around the mid-height region. This difference can be attributed mainly to manufacturing tolerances introduced during laser cutting and assembly, which could have caused local geometric imperfections and shifted the onset of deformation.

The simulated force–displacement curve showed reasonable agreement with the experimental response. Figure 9b compares the finite element prediction with the force–displacement curves obtained from all three experimental replicates. The model reproduced the overall force evolution of the Oval-Hybrid tube. The experimental curves exhibited abrupt force drops between approximately 7 and 9 mm displacement, which were associated with local fracture around the edges of the laser-cut perforations. The precise magnitude of these drops varied among the experimental replicates, reflecting their sensitivity to specimen-specific local imperfections. The finite element model employed idealised geometry and did not include a material fracture or damage criterion. It was developed to reproduce the global elastoplastic crushing response and principal deformation mode. Consequently, the numerical curve remained smoother than the experimental curves. Nevertheless, the model captured the overall force evolution and principal deformation characteristics with acceptable agreement, supporting its use for the subsequent comparative Oval-Hybrid parametric analysis.

Quantitative agreement between the experimental and numerical responses was assessed using the maximum force, MCF, CFE, and EA, as summarised in Table 4. The finite element model predicted a maximum force of 96.18 kN, an MCF of 80.82 kN, a CFE of 84.03%, and an EA of 1.212 kJ. Relative to the corresponding experimental mean values, the differences in maximum force, MCF, CFE, and EA were −3.87%, −1.31%, +2.70%, and −1.38%, respectively. The maximum absolute relative difference among the directly comparable force- and energy-based quantities was therefore below 4%. Together with the deformation-mode and force–displacement comparisons in Figure 9, these results support the use of the model for the subsequent sensitivity analyses within the Oval-Hybrid configuration.

## 6. Parametric Analysis of Oval-Hybrid Tubular Metamaterials

### 6.1. Effect of Oval-Hole Aspect Ratio

**Figure 10:**
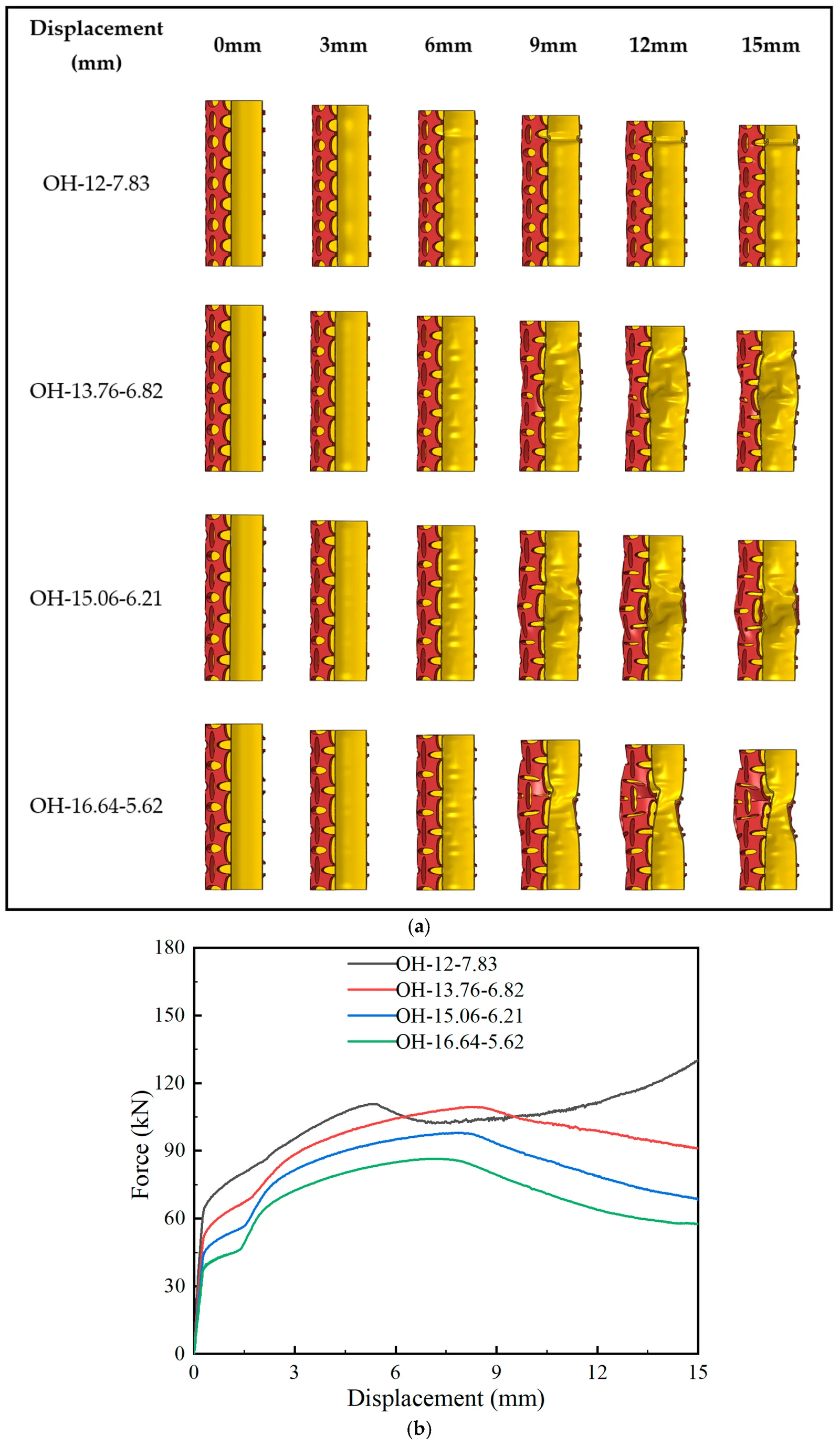
Abaqus/explicit finite element results for Oval-Hybrid tubes with different oval-hole aspect ratios: (**a**) numerically predicted deformation modes; and (**b**) numerical force–displacement curves. The perforated stainless-steel outer tube and aluminium inner tube are shown in red and yellow, respectively.

To investigate the effect of oval-hole aspect ratio on the mechanical response of Oval-Hybrid tubular metamaterials, four models with different combinations of major axis a and minor axis b were designed while maintaining a nearly constant porosity. The wall thickness, height, and diameters of the inner and outer tubes were kept unchanged to ensure comparable structural configurations. The four configurations were denoted as OH-12-7.83, OH-13.76-6.82, OH-15.06-6.21, and OH-16.64-5.62, where the two numbers represent the values of a and b, respectively. The oval-hole aspect ratio was defined as (R = a/b). The corresponding oval-hole dimensions, porosity values, and aspect ratios are listed in Table 5, and the deformation modes and force–displacement responses are shown in Figure 10.

The force–displacement curves in Figure 10b show that the deformation response was strongly affected by the oval-hole aspect ratio. The OH-12-7.83 tube, with the smallest R of 1.53, retained its global axial alignment and developed a comparatively progressive local folding response. After reaching an initial peak force of 110.68 kN, its load temporarily decreased and subsequently recovered, reaching a maximum force of 130.29 kN near the end of the prescribed displacement. By contrast, the OH-13.76-6.82, OH-15.06-6.21, and OH-16.64-5.62 tubes exhibited progressive post-peak force reductions and increasingly pronounced asymmetric global bending. These results indicate that increasing R reduced the ability of the perforated outer tube to maintain progressive collapse and sustained absolute load-bearing capacity within the investigated fixed-porosity series.

The quantitative results in Table 6 show that the performance trend depended on the selected metric. As R increased from 1.53 to 2.96, *F*_max_ decreased from 130.29 to 86.45 kN, MCF decreased from 102.63 to 69.43 kN, EA decreased from 1.54 to 1.04 kJ, and SEA decreased from 7.05 to 4.81 kJ/kg. CFE varied non-monotonically and reached its highest value of 85.70% for OH-13.76-6.82 at R = 2.02, compared with 78.77% for OH-12-7.83 at R = 1.53. A smaller R therefore favoured progressive folding, absolute load-bearing capacity, and total energy absorption, while an intermediate R provided the highest force-retention efficiency. The preferred aspect ratio depends on the design objective within the investigated fixed-porosity series.

Interaction energy (IE) was used as a residual energy term associated with the coupled response of the inner and outer tubes. It was calculated as the difference between the total energy absorbed by the hybrid tube and the sum of the energy contributions of the inner and outer tubes extracted from the same hybrid finite element model:

(7) $$IE=EA-{EA}_{o}-{EA}_{i}$$

where EA is the total energy absorbed by the hybrid tube, EA_o_ is the energy absorbed by the perforated outer tube, and EA_i_ is the energy absorbed by the aluminium inner tube. IE therefore represents the non-additive energy contribution under the adopted energy partition.

As shown in Table 7, IE ranged from 0.026 to 0.047 kJ and represented only 2.20–3.05% of the total EA. Although its absolute value decreased by approximately 44.7% as R increased from 1.53 to 2.96, its fractional contribution remained modest for all four configurations. The small IE values show that the additional interaction-energy contribution was limited. The main effect of combining the two tubes was reflected in the changes in deformation mode, load transfer, and energy distribution between the inner and outer tubes.

The energy distribution shifted markedly with increasing aspect ratio. The energy absorbed by the outer tube decreased from 1.072 to 0.453 kJ, and its contribution to the total EA decreased from 69.656% to 43.516%. In contrast, the energy absorbed by the inner tube increased from 0.420 to 0.562 kJ, and its contribution increased from 27.290% to 53.987%. Meanwhile, the total EA decreased from 1.539 to 1.041 kJ. These results indicate that the smaller-aspect-ratio configuration maintained a greater load-bearing and energy-dissipation contribution from the perforated outer tube, whereas increasing the aspect ratio progressively shifted the energy-absorption contribution towards the inner aluminium tube.

### 6.2. Effect of Inner-Tube Wall Thickness

To investigate the effect of inner-tube wall thickness on the mechanical response of Oval-Hybrid tubes, four models with different aluminium inner-tube wall thicknesses were designed while keeping the oval-hole geometry of the outer tube unchanged. The dimensions of the inner and outer tubes are listed in Table 8. The wall thickness of the outer tube, the height of both tubes, the outer and inner diameters of the outer tube, and the outer diameter of the inner tube were kept constant.

To quantify the stiffness relationship between the two tube components, a nominal inner-to-outer axial-rigidity ratio was introduced. The cross-sectional area of the inner tube was calculated from its outer and inner diameters, while the mean material area of the perforated outer tube was calculated from its remaining CAD solid volume divided by the tube length:

(8) $$\eta_{K}\;=\;\frac{K_{i}}{K_{o}}\;=\frac{E_{i}A_{i}}{\;E_{o}A_{o,eff}}$$

(9) $$A_{i}=\frac{\pi }{4}({D_{o,i}}^{2}-{D_{i,i}}^{2})$$

(10) $$A_{o,eff}=\frac{V_{p}}{L}$$

where $E_{i}$ and $E_{o}$ are the measured Young’s moduli of the aluminium inner tube and stainless-steel outer tube, respectively; $A_{i}$ is the cross-sectional area of the inner tube; $A_{o,eff}$ is the mean material area of the perforated outer tube; $D_{o,i}$ and $D_{i,i}$ are the outer and inner diameters of the aluminium inner tube; $V_{p}$ is the remaining CAD solid volume of the perforated Oval outer tube; and L is the tube length. Based on $E_{i}$ = 73.71 GPa, $E_{o}$ = 193 GPa, $V_{p}$ = 22,795.322 mm^3^, and L = 100 mm, the nominal axial-rigidity ratios were 0.112, 0.221, 0.328, and 0.432 for inner-tube wall thicknesses of 0.5, 1.0, 1.5, and 2.0 mm, respectively. This nominal ratio was used to compare the relative axial rigidity of the inner and outer tubes across the four wall-thickness configurations.

**Figure 11:**
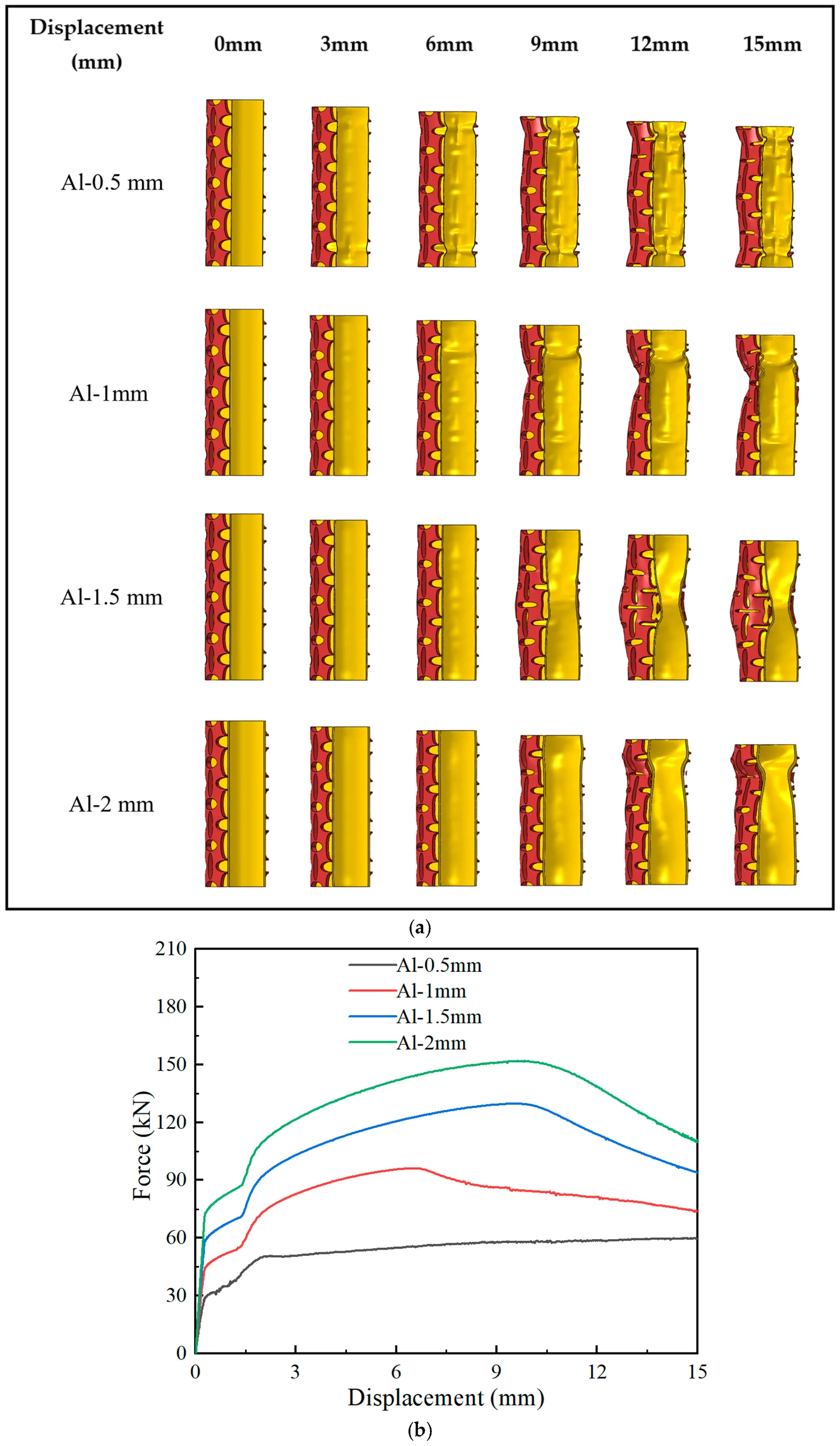
Abaqus/Explicit finite element results for Oval-Hybrid tubes with different inner-tube wall thicknesses: (**a**) numerically predicted deformation modes; and (**b**) numerical force–displacement curves. The perforated stainless-steel outer tube and aluminium inner tube are shown in red and yellow, respectively.

The deformation sequences and force–displacement responses of the Oval-Hybrid tubes with different inner-tube wall thicknesses are shown in Figure 11. For the Al-0.5 mm configuration, the nominal rigidity ratio was 0.112 and the relatively compliant inner tube allowed comparatively progressive local folding without pronounced global lateral deflection, producing a stable force response. For the Al-1 mm, Al-1.5 mm, and Al-2 mm configurations, the ratios increased to 0.221, 0.328, and 0.432, respectively. The stronger inner support increased the peak load but progressively concentrated the deformation and produced more localised or asymmetric buckling followed by post-peak force reduction. The observed transition was associated with the increase in the relative axial rigidity of the inner tube.

As shown in Table 9, increasing the inner-tube wall thickness from 0.5 to 2.0 mm increased *P*_initial_ from 50.78 to 151.94 kN, *F*_max_ from 59.94 to 151.94 kN, MCF from 53.61 to 128.92 kN, EA from 0.80 to 1.93 kJ, and SEA from 4.23 to 8.00 kJ/kg. However, CFE varied non-monotonically: the 0.5 mm tube achieved the highest value of 89.43%, whereas the values for the 1.0, 1.5, and 2.0 mm tubes were 84.03%, 83.72%, and 84.85%, respectively. Increasing thickness therefore improved absolute load-bearing and energy absorption but did not improve force-retention efficiency monotonically.

Table 10 shows that total EA increased from 0.804 to 1.934 kJ as the inner-tube thickness increased. The inner-tube contribution increased from 0.252 to 1.073 kJ and from 31.34% to 55.48% of total EA. The absolute outer-tube contribution also increased from 0.516 to 0.820 kJ, but its fractional contribution decreased from 64.18% to 42.40%. IE remained modest at 0.029–0.041 kJ, corresponding to only 1.78–4.48% of total EA. Thus, the main wall-thickness effect was a redistribution of load and energy absorption towards the inner aluminium tube rather than a large additional interaction-energy term.

Overall, increasing the inner-tube wall thickness enhanced the absolute load-bearing capacity, EA, and SEA of the Oval-Hybrid tube, while the 0.5 mm configuration retained the highest CFE. The increase in the nominal inner-to-outer axial-rigidity ratio was accompanied by more localised or asymmetric buckling. The four investigated cases indicate a clear stiffness-related trend but are insufficient to identify a single stiffness-matching threshold.

## 7. Conclusions

This study experimentally and numerically investigated the effects of perforation topology and inner-tube support on the early-stage axial crushing response of bio-inspired hybrid tubes. The main conclusions are:

1. Perforation topology strongly affected collapse stability. The Oval-Single and Square-Single tubes exhibited relatively stable deformation, whereas the Circle-Single and Re-entrant-Single tubes were more susceptible to local buckling and post-peak load reduction.
2. Adding an aluminium inner tube improved load transfer and energy absorption. The Circle-Hybrid tube achieved the highest *F*_max_, MCF, and mean SEA of 6.95 ± 0.04 kJ/kg. The Oval-Hybrid tube achieved the second-highest mean SEA of 5.76 ± 0.25 kJ/kg, while its *F*_max_ was 24.08% lower and its CFE was 6.68 percentage points higher than those of the Circle-Hybrid tube.
3. Within the fixed-porosity Oval-Hybrid sensitivity study, decreasing the oval-hole aspect ratio from 2.96 to 1.53 increased *F*_max_ from 86.45 to 130.29 kN and EA from 1.04 to 1.54 kJ. However, the highest CFE of 85.70% occurred at the intermediate aspect ratio of 2.02, indicating that the preferred aspect ratio depends on the selected performance metric. Increasing the inner-tube wall thickness from 0.5 to 2.0 mm increased *P*_initial_ from 50.78 to 151.94 kN, *F*_max_ from 59.94 to 151.94 kN, EA from 0.80 to 1.93 kJ, and SEA from 4.23 to 8.00 kJ/kg. The 0.5 mm tube retained the highest CFE of 89.43%, while deformation became more localised or asymmetric as the inner-tube thickness increased.

Overall, perforation topology and inner-tube support can be tailored to balance peak force, crushing efficiency, deformation stability, and energy absorption.

## Figures and Tables

**Table 1:** (**a**) Dimensions of all tube samples (mm). (**b**) CAD solid volumes, material-removal porosities, and measured masses of the tested tube configurations.

(**a**)						
**Specimens**	**Component**	**Exterior Diameter**	**Interior Diameter**	**Wall Thickness**	**Length**	
Aluminium tube	Single tube	43.03 ± 0.03	40.83 ± 0.03	1.10 ± 0.03	99.87 ± 0.03	
Oval-Single	Single tube	49.87 ± 0.05	43.81 ± 0.05	3.03 ± 0.03	99.87 ± 0.03	
Circle-Single	Single tube	49.87 ± 0.05	43.80 ± 0.05	3.03 ± 0.03	99.87 ± 0.03	
Square-Single	Single tube	48.60 ± 0.05	44.00 ± 0.05	2.30 ± 0.03	99.87 ± 0.03	
Re-entrant-Single	Single tube	49.40 ± 0.05	44.00 ± 0.05	2.70 ± 0.03	99.87 ± 0.03	
Oval-Hybrid	Outer tube	49.87 ± 0.05	43.81 ± 0.05	3.03 ± 0.03	99.87 ± 0.03	
	Inner tube	43.03 ± 0.03	40.83 ± 0.05	1.10 ± 0.01	99.87 ± 0.01	
Circle-Hybrid	Outer tube	49.87 ± 0.05	43.80 ± 0.05	3.03 ± 0.03	99.87 ± 0.03	
	Inner tube	43.03 ± 0.03	40.86 ± 0.03	1.07 ± 0.01	99.87 ± 0.01	
Square-Hybrid	Outer tube	48.60 ± 0.05	44.00 ± 0.05	2.30 ± 0.03	99.87 ± 0.03	
	Inner tube	43.03 ± 0.03	40.86 ± 0.03	1.07 ± 0.01	99.87 ± 0.01	
Re-entrant-Hybrid	Outer tube	49.40 ± 0.05	44.00 ± 0.05	2.70 ± 0.03	99.87 ± 0.03	
	Inner tube	43.03 ± 0.03	40.86 ± 0.03	1.07 ± 0.01	99.87 ± 0.01	
(**b**)						
**Configuration**	**CAD Solid Volume (mm^3^)**	**Material-Removal** **Porosity (%)**	**S1** **(kg)**	**S2** **(kg)**	**S3** **(kg)**	**Mean ± SD** **(kg)**
Aluminium tube	N/A	0.00	0.0368	0.0355	0.0366	0.0363 ± 0.0007
Oval-Single	22,795.32	48.54	0.1731	0.1768	0.1757	0.1752 ± 0.0019
Circle-Single	22,784.78	48.56	0.1759	0.1791	0.1775	0.1775 ± 0.0016
Square-Single	22,803	31.84	0.1686	0.1663	0.1685	0.1678 ± 0.0013
Re-entrant-Single	22,777	42.50	0.1731	0.1727	0.1708	0.1722 ± 0.0012
Oval-Hybrid	22,795.32	48.54	0.2120	0.2135	0.2147	0.2134 ± 0.0014
Circle-Hybrid	22,784.78	48.56	0.2116	0.2152	0.2146	0.2138 ± 0.0019
Square-Hybrid	22,803	31.84	0.2036	0.2009	0.2027	0.2024 ± 0.0014
Re-entrant-Hybrid	22,777	42.50	0.2079	0.2108	0.2098	0.2095 ± 0.0015

Note: CAD solid volume and material-removal porosity were calculated from the nominal CAD geometry of the perforated outer tube. The measured dimensions in Table 1a were not used in this calculation. For hybrid specimens, mass refers to the complete tested configuration. S1–S3 denote the three independently tested specimens. N/A indicates “not applicable”; CAD solid volume is reported only for the perforated outer tube.

**Table 2:** Mechanical properties of aluminium alloy 6063-T5 and stainless steel 304.

Material	Density (kg/m^3^)	Young’s Modulus (GPa)	Poisson’s Ratio	Yield Stress (MPa)	Ultimate Strength (MPa)
6063-1 mm aluminium	2700	73.71	0.3	147.42	182.84
304-3 mm steel	7930	193	0.3	331.07	764.83

**Table 4:** Quantitative comparison between the experimental results and finite element prediction for the Oval-Hybrid tube.

Metric	Experiment	FEM (19.80 mm/min)	Difference
*F*_max_ (kN)	100.05 ± 2.31	96.18	−3.87%
MCF (kN)	81.89 ± 3.88	80.82	−1.31%
CFE (%)	81.82 ± 2.03	84.03	+2.70%
EA (kJ)	1.229 ± 0.058	1.212	−1.38%

Note: Experimental values are reported as the mean ± standard deviation of three independent tests (*n* = 3), whereas the FEM values are deterministic predictions. Differences were calculated from the values reported in the table as (FEM − experimental mean)/experimental mean × 100%.

**Table 5:** Dimensions and aspect ratios of oval holes in Oval-Hybrid tubes.

Models	a (mm)	b (mm)	P (%)	R (a/b)
OH-12-7.83	12	7.83	48.31	1.53
OH-13.76-6.82	13.76	6.82	48.33	2.02
OH-15.06-6.21	15.06	6.21	48.33	2.43
OH-16.64-5.62	16.64	5.62	48.31	2.96

**Table 6:** Mechanical performance of Oval-Hybrid tubes with different oval-hole aspect ratios.

Type	*P*_initial_ (kN)	*F*_max_ (kN)	MCF (kN)	CFE (%)	EA (kJ)	SEA (kJ/kg)
OH-12-7.83	110.68	130.29	102.63	78.77	1.54	7.05
OH-13.76-6.82	109.50	109.50	93.84	85.70	1.41	6.46
OH-15.06-6.21	97.96	97.96	80.79	82.48	1.21	5.35
OH-16.64-5.62	86.45	86.45	69.43	80.31	1.04	4.81

**Table 7:** Energy distribution in Oval-Hybrid tubes with different oval-hole aspect ratios.

Type	EA_i_ (kJ)	EA_o_ (kJ)	Total (kJ)	IE (kJ)	EA_i_ (%)	EA_o_ (%)	IE (%)
OH-12-7.83	0.420	1.072	1.539	0.047	27.290	69.656	3.054
OH-13.76-6.82	0.557	0.820	1.408	0.031	39.560	58.239	2.202
OH-15.06-6.21	0.564	0.618	1.212	0.030	46.535	50.990	2.475
OH-16.64-5.62	0.562	0.453	1.041	0.026	53.987	43.516	2.498

Note: EA_i_, EA_o_, and IE denote the energy absorbed by the aluminium inner tube, the perforated outer tube, and the interaction-energy term, respectively. All percentages were calculated relative to the total EA. Because the values are rounded to three decimal places, a row total may differ from 100.000% by 0.001 percentage points.

**Table 8:** Dimensions of Oval-Hybrid tubes with different inner-tube wall thicknesses (mm).

Specimens	Component	Exterior Diameter	Interior Diameter	Wall Thickness	Length
Al-0.5 mm	Outer tube	50	44	3	100
	Inner tube	43	42	0.5	100
Al-1 mm	Outer tube	50	44	3	100
	Inner tube	43	41	1	100
Al-1.5 mm	Outer tube	50	44	3	100
	Inner tube	43	40	1.5	100
Al-2 mm	Outer tube	50	44	3	100
	Inner tube	43	39	2	100

**Table 9:** Mechanical performance of Oval-Hybrid tubes with different inner-tube wall thicknesses.

Type	*P*_initial_ (kN)	*F*_max_ (kN)	MCF (kN)	CFE (%)	EA (kJ)	SEA (kJ/kg)
Al-0.5 mm	50.78	59.94	53.61	89.43	0.80	4.23
Al-1 mm	96.18	96.18	80.82	84.03	1.21	5.78
Al-1.5 mm	129.80	129.80	108.66	83.72	1.63	7.24
Al-2 mm	151.94	151.94	128.92	84.85	1.93	8.00

**Table 10:** Energy distribution in Oval-Hybrid tubes with different inner-tube wall thicknesses.

Type	EA_i_ (kJ)	EA_o_ (kJ)	Total (kJ)	IE (kJ)	EA_i_ (%)	EA_o_ (%)	IE (%)
Al-0.5 mm	0.252	0.516	0.804	0.036	31.34	64.18	4.48
Al-1 mm	0.509	0.662	1.212	0.041	42.00	54.62	3.38
Al-1.5 mm	0.858	0.743	1.630	0.029	52.64	45.58	1.78
Al-2 mm	1.073	0.820	1.934	0.041	55.48	42.40	2.12

**Figure A1:**
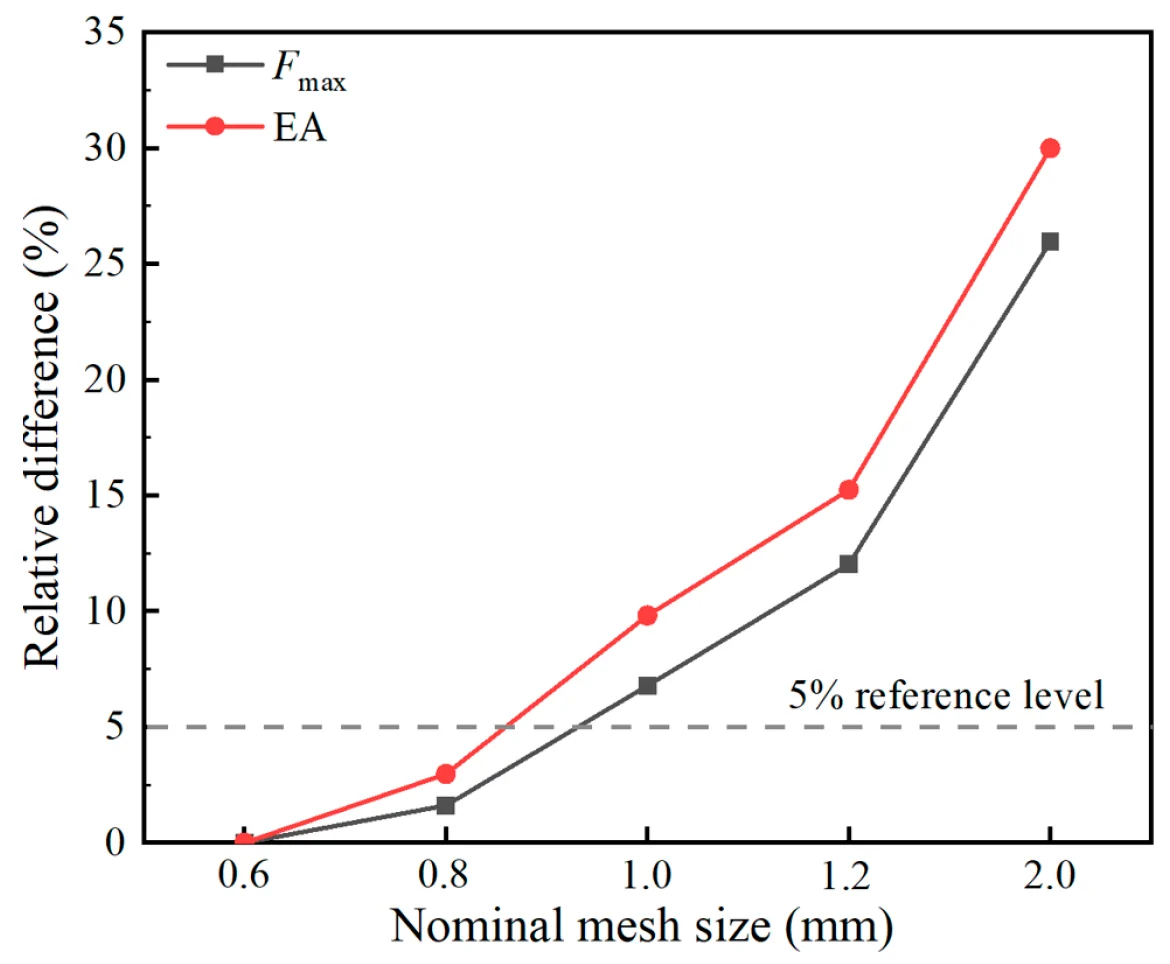
Relative differences in predicted maximum force ($F_{max}$) and energy absorption (EA) for five nominal mesh sizes, using the 0.6 mm mesh as the refinement reference. The dashed line indicates the 5% reference level.

**Figure A2:**
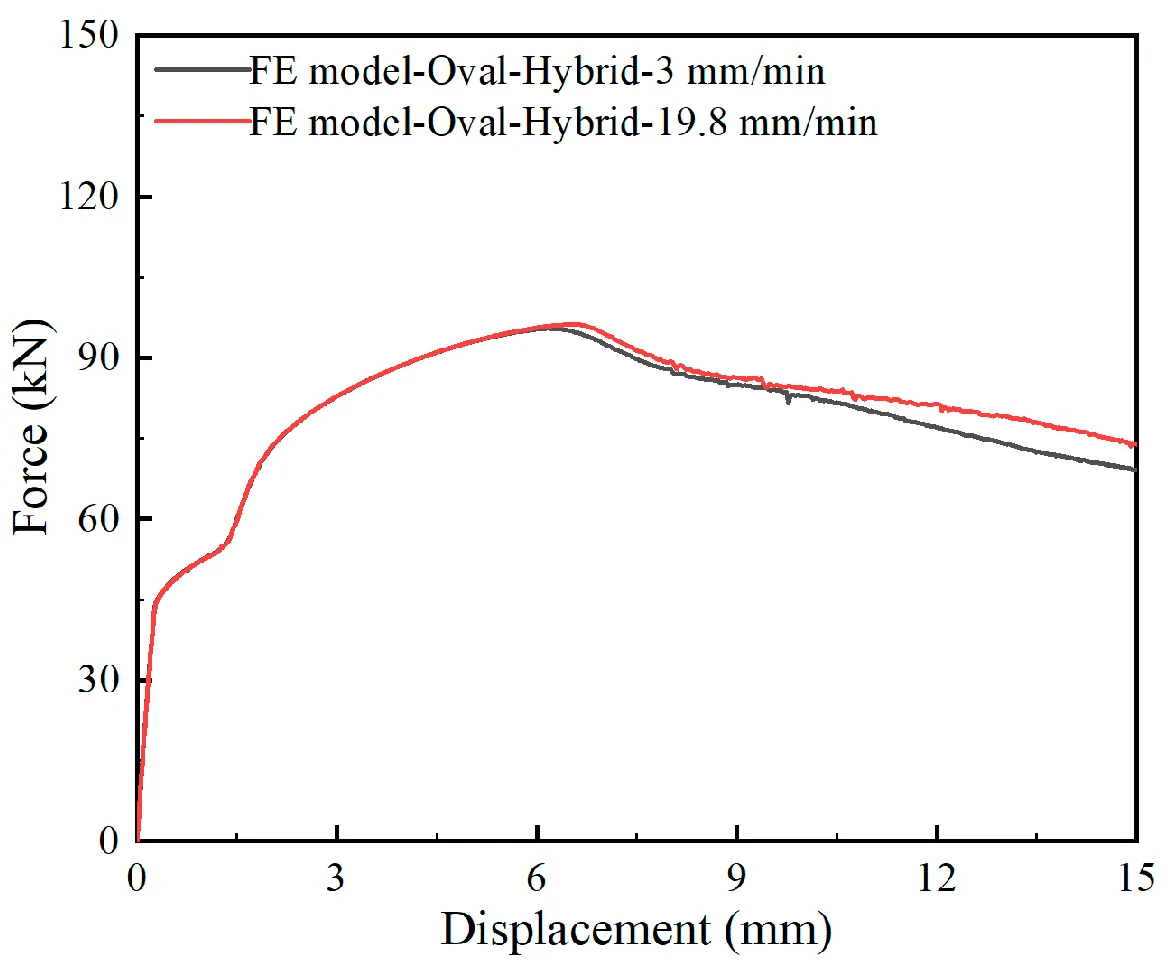
Comparison of the numerical force–displacement responses obtained using prescribed displacement rates of 3.0 and 19.80 mm/min.

## Data Availability

The data supporting the findings of this study are available from the corresponding author upon reasonable request.

## Appendix A. Supplementary Numerical Modelling Details

### Appendix A.1. Mesh Convergence

Nominal mesh sizes of 2.0, 1.2, 1.0, 0.8, and 0.6 mm gave total node/element counts of 23,232/39,009, 65,875/136,007, 97,177/209,305, 155,830/365,966, and 302,968/810,841, respectively, for the two deformable tube components. Relative to the 0.6 mm mesh, the 0.8 mm mesh differed by 1.61% in maximum force and 2.97% in energy absorption up to a displacement of 15 mm, while reducing the total element count by 54.87%. At the adopted nominal mesh size of 0.8 mm, the outer tube contained 72,166 nodes and 303,716 C3D4 elements, while the inner tube contained 83,664 nodes and 62,250 C3D8R elements. The 0.8 mm mesh was therefore adopted for model validation and the subsequent parametric simulations.

### Appendix A.2. Loading-Rate Verification

To reduce computational cost, the prescribed displacement was applied at 19.80 mm/min, compared with 3.0 mm/min in the experiments. Based on the nominal initial tube length of 100 mm, these velocities correspond to nominal axial strain rates of $3.3\times 10^{-3}$ and $5.0\times 10^{-4}\;s^{-1}$, respectively. The material input was rate independent and was derived from the quasi-static tensile tests. To verify that the numerical acceleration did not materially alter the predicted global response, an otherwise identical simulation was performed at the experimental loading rate of 3.0 mm/min. As shown in Figure A2, the two numerical force–displacement curves were nearly coincident through the initial loading and peak-force regions and showed only a small post-peak separation. In addition, the ratio of kinetic energy to internal energy remained below 5% throughout the simulation performed at 19.80 mm/min. The agreement between the two numerical responses, together with the energy-ratio check, supports the use of 19.80 mm/min for the present quasi-static finite element calculations under the adopted rate-independent constitutive formulation.
